# Sesquiterpene Lactones with the 12,8-Guaianolide Skeleton from Algerian *Centaurea omphalotricha*

**DOI:** 10.3390/biom11071053

**Published:** 2021-07-18

**Authors:** Sara Kebbi, Maria Letizia Ciavatta, Ali Mokhtar Mahmoud, Marianna Carbone, Alessia Ligresti, Ramdane Seghiri, Margherita Gavagnin

**Affiliations:** 1 Consiglio Nazionale delle Ricerche (CNR), Istituto di Chimica Biomolecolare (ICB), Via Campi Flegrei, 34-80078 Pozzuoli, Italy; sara.kebbi@umc.edu.dz (S.K.); amokhtar@icb.cnr.it (A.M.M.); mcarbone@icb.cnr.it (M.C.); aligresti@icb.cnr.it (A.L.); mgavagnin@icb.cnr.it (M.G.); 2 Unit of Valorization of Natural Resources, Bioactive Molecules and Physicochemical and Biological Analyzes (VARENBIOMOL), Department of Chemistry, Faculty of Exact Sciences, University of Mentouri Brothers Constantine 1, Constantine 25000, Algeria; seghiri25000@yahoo.fr

**Keywords:** plant secondary metabolites, sesquiterpene lactones, Asteraceae, spectroscopic methods

## Abstract

In continuing our investigation on the chemical diversity of Algerian plants, we examined *Centaurea omphalotricha*, whose chemical composition has been poorly studied. The present work was aimed at characterizing the secondary metabolite pattern of the CHCl_3_ extract of the aerial parts of this plant that displayed antiproliferative properties in a preliminary screening on HeLa cell line. The chemical analysis led us to characterize the bioactive oxygenated terpenoid fraction which includes, within major known metabolites, two new minor sesquiterpene lactones, centaurolide-A (**1**) and centaurolide-B (**2**). The structures of two compounds exhibiting the 12,8-guaianolide skeleton were determined by spectroscopic methods as well as by chemical correlation with inuviscolide (**3**), a well-known bioactive guaianolide isolated from *Dittrichia* (=*Inula*) *viscosa*. Centaurolides A and B represent the first report of 8,12-guaianolide skeleton in *Centaurea* genus. The effect of new compounds **1** and **2** and inuviscolide (**3**) on HeLa cell has also been evaluated.

## 1. Introduction

The genus *Centaurea* (family Asteraceae, tribe Centaureinae) includes more than 500 species, distributed all around the world and prevalently located in Western Asia and the Mediterranean region [1]. In particular, North Africa is an important center for endemisms of *Centaurea* taxa with several species narrowly distributed in restricted regions. Algerian flora comprises forty-five species of *Centaurea*, seven of which are growing in the Sahara [2]. Many species of this genus have been used in folk medicine as antipyretic, antibacterial, anti-inflammatory, and antiproliferative agents for the treatment of various ailments [3,4]. Phytochemical studies carried out on different *Centaurea* species have evidenced that compounds responsible for their pharmacological properties are mainly sesquiterpene lactones, including eudesmanolides and guaianolides [4,5]. In the course of our ongoing studies on Algerian medicinal plants [6,7,8,9,10,11], we have analyzed the chemical composition of *Centaurea omphalotricha* Coss. and Durieu ex Batt.etTrab., a plant native both to Algeria and Tunisia, growing especially in desertic areas. In particular, our study was carried out on a specimen collected during the flowering phase in May 2017 from Bousaada, northeast Algeria. Previous chemical studies on *C. omphalotricha* were reported only from plants collected in Algeria, in areas different from our collection site, and describe the chemical composition of flavonoid and triterpenoidic fraction [12] as well as sesquiterpene lactone content [13]. Here, we present the results of our investigation on the CHCl_3_ soluble portion of the hydroalcoholic extract of the whole aerial part of the plant that resulted to be active in a preliminary antiproliferative assay on HeLa cell lines. This study was aimed at characterizing the chemical diversity of Algerian *C. omphalotricha* by comparing the secondary metabolite pattern of our sample with literature data for this species, in particular, targeting the sesquiterpene components.

A series of compounds belonging to different structural groups including sesquiterpenoids and polyphenols was identified in the extract. Among these, two new minor compounds, centaurolide-A (**1**) and -B (**2**), with the 12,8-guaianolide framework that has never been reported from the genus *Centaurea* to date, were isolated. The structural characterization of compounds **1** and **2** was achieved by spectroscopic methods as well as by chemical transformation of structurally related inuviscolide (**3**) [14,15,16], isolated from *Dittrichia* (ex *Inula*) *viscosa*, opportunely collected in Molise (Italy) in the course of this study. The results of HeLa cell antiproliferative assay of compounds **1**–**3** have been also discussed.

## 2. Materials and Methods

### 2.1. General Experimental Procedures

Optical rotations were measured on a Jasco P-2000digital polarimeter. ESIMS were performed on a Micromass Q-TOF MicroTM coupled with a HPLC Waters Alliance 2695. The instrument was calibrated by using a PEG mixture from 200 to 1000 MW (resolution specification 5000 FWHM, deviation <5 ppm RMS in the presence of a known lock mass). High-resolution mass spectra (HRESIMS) were acquired on a Q-Exactive hybrid quadrupole-orbitrap mass spectrometer (Thermo Scientific). NMR experiments were recorded at the ICB-NMR Service Centre. Chemical shifts values are reported in ppm and referenced to internal signals of residual protons (CDCl_3_, ^1^H δ 7.26, ^13^C 77.0 ppm; C_6_D_6_, ^1^H δ 7.15, ^13^C 128.0 ppm, CD_3_OD, ^1^H δ 3.34, ^13^C 49.0 ppm). 1D and 2D NMR spectra were acquired on a Bruker Avance-400 spectrometer using an inverse probe fitted with a gradient along the *Z*-axis, on a Bruker Avance III HD spectrometer equipped with a CryoProbe Prodigy, and on a DRX 600 spectrometer (600 MHZ for 1H, 150 MHz for ^13^C) equipped with a three-channels inverse (TCI) CryoProbe. HPLC was performed on a Jasco system (PU4180 pump equipped with a Jasco UV/Vis detector PU4075) using a semipreparative column (C18 Ascentis, Supelco, 250 × 10 mm). Silica-gel chromatography was performed using pre-coated Merck F254 plates (TLC) and Merck Kieselgel 60 powder (70–230 mesh). The spots on TLC were visualized under UV light (254 nm) and then spraying with 10% H_2_SO_4_ in water followed by heating.

### 2.2. Plant Material

The whole aerial parts (flowers, leaves, and epigeal portion) of *Centaurea omphalotricha* were collected during the flowering phase in May 2017 from Bousaada, eastern Algeria. The plant was authenticated by Dr. D. Sarri (Biology Department, University of Mohammed Boudiaf-M’Sila, Algeria). A voucher specimen (CO19/05/17) has been deposed at the Herbarium of the VARENBIOMOL Research Unit, University des Frères Mentouri, Constantine, Algeria. The aerial parts of *Dittrichia* (=*Inula*) *viscosa* were collected during August 2020 in Lucito (loc. Titolo, Molise, Italy). The plant was authenticated by Prof. V. De Feo (University of Fisciano, Salerno, Italy). A voucher specimen (code INU-2020) is stored at ICB.

### 2.3. Extraction and Isolation of Secondary Metabolites from C. omphalotricha

Air-dried aerial parts of *C. omphalotricha* (1600 g) were macerated with a hydroalcoholic solution of MeOH/H_2_O (8:2, *v*/*v*) for 48 h at room temperature three times. After filtration, the organic solvent was evaporated to give a crude residue, which was suspended in water and successively extracted with CHCl_3_, then EtOAc and finally *n*-butanol. The organic phases were concentrated to give the corresponding extracts: CHCl_3_ (4.38 g), EtOAc (5.77 g) and *n*-butanol (25 g), respectively. A portion (3.3 g) of the CHCl_3_ extract was fractionated by silica-gel column chromatography by eluting first with a gradient of CH_2_Cl_2_ in petroleum ether (PE), and, subsequently, with a gradient of acetone in CH_2_Cl_2_ to obtain 43 fractions (C1–C43). Nine selected fractions (C13, C16, C19, C24, C27, C29, C30, C34, C37) were taken into consideration after TLC chromatography analysis and preliminary ^1^H-NMR inspection. Purification steps of fractions C13, C16, C19, C24, C27, C30, C34, and C37 to obtain pure known compounds are described in detail in the Supplementary Materials. Fraction C29 (200 mg) was loaded onto a silica-gel (7.0 g) column packed in PE and eluted initially with an increasing gradient of Et_2_O in PE (10:0, 9:1, 8:2, 7:3, 6:4, 5:5, 4:6, 3:7, 2:8, 1:9, 0:10), then with CHCl_3_, and finally with MeOH, affording 13 subfractions C29-1–C29-13. Subfraction C29-3 (8.0 mg) was further purified by 10% AgNO_3_ silica-gel pipette Pasteur, using as eluent system a mixture of PE/CHCl_3_ 1:1, giving centaurolide A (**1**, 1.6 mg), and centaurolide B (**2**, 1.5 mg) in pure form. Purification of remaining subfractions from C29 column chromatographic separation (C29-6, C29-7, C29-8, C29-10) is reported in the Supplementary Materials.

#### 2.3.1. Centaurolide A (**1**)

Colorless oil; Rf 0.45 (PE/Et_2_O 1:1); [α]_D_^25^ -39.3 (c, 0.16, CHCl_3_); UV (MeOH) λ_max_ (log ε) 204 (3.65) nm; ECD (MeOH) 208 Δε (−1.95); IR (KBr) ν_max_ 3387, 2924, 2852, 1765, 1671, 1262, 1162, 1005, 752 cm^−1^; ^1^H and ^13^C NMR see Table 1 and Table 2; HR-ESI-MS *m*/*z* 253.1199 (Calcd. For C_15_H_18_O_2_Na, 253.1204).

#### 2.3.2. Centaurolide B (**2**)

Colorless oil; R*f* 0.45 (PE/Et_2_O 1:1); [α]_D_^25^ -25.5 (c, 0.06, CHCl_3_); UV (MeOH) λ_max_ (log ε) 205 (4.14) nm; ECD (MeOH) 208 Δε (−1.75); IR (KBr) ν_max_ 3414, 2923, 2852, 1769, 1634, 1383, 1256, 1006, 804 cm^−1^; ^1^H and ^13^C NMR in CDCl_3_ see Table 1 and Table 2; HR-ESI-MS *m*/*z* 253.1197 (Calcd. For C_15_H_18_O_2_Na, 253.1204); ^1^H NMR data (C_6_D_6_, 600 MHz): δ 6.08 (1H, br d, *J* = 3.6 Hz, H-13a), 5.26 (1H, br s, H-3), 4.87 (1H, s, H-14a), 4.84 (1H, br d, *J* = 3.1 Hz, H-13b), 4.74 (1H, s, H-14b), 3.67 (1H, ddd, *J* = 11.1, 9.3, 4.5 Hz, H-8), 2.96 (1H, br dd, *J* = 15.9, 4.5 Hz, H-9a), 2.33 (1H, m, H-2a), 2.32 (1H, m, H-1), 2.27 (1H, br dd, *J* = 15.9, 11.1 Hz, H-9b), 2.05 (1H, m, H-2b), 1.96 (1H, ddd, *J* = 12.1, 9.3, 3.1 Hz, H-7), 1.87 (1H, m, H-5), 1.81 (1H, ddd, *J* = 12.9, 3.1, 2.8 Hz, H-6a), 1.43 (3H, Br s, H_3_-15), 0.50 (1H, ddd, *J* = 12.1, 12.1, 12.1 Hz, H-6b); ^13^C NMR data (C_6_D_6_, 150 MHz): δ 170.8 (C = O, C-12), 145.5 (C, C-10), 140.8 (C, C-11), 140.7 (C, C-4), 124.2 (CH, C-3), 118.4 (CH_2_, C-13), 111.3 (CH_2_, C-14), 80.8 (CH, C-8),54.3 (CH, C-5), 47.6 (CH, C-7), 40.6 (CH_2_, C-9), 34.7 (CH_2_, C-2), 32.6 (CH_2_, C-6), 14.8 (CH_3_, C-15).

### 2.4. Extraction and Isolation of Inuviscolide from D. viscosa

Air-dried powdered aerial parts (30 g) of *D. viscosa* were macerated with a hydroalcoholic solution of EtOH/H_2_O (8:2, *v*/*v*, 300 mL × 3) at room temperature. After filtration, the organic solvent was combined and evaporated to give a residue that was suspended in water and subsequently extracted with CHCl_3_, EtOAc and finally n-butanol. The organic phases from these partitions were concentrated to give the corresponding CHCl_3_ (3.0 g), EtOAc (0.570 g) and *n*-butanol (0.900 g) extracts, respectively. The CHCl_3_ extract was loaded as a slurry into a silica-gel column (SiO_2_, 100 g) packed in light petroleum ether (PE)/diethyl ether (Et_2_O), 7:3, and eluted with a gradient of Et_2_O in PE (7:3, 1:1, 4:6, 3:7, 2:8), Et_2_O (100%), CHCl_3_(100%), CHCl_3_/MeOH 8:2, MeOH (100%), giving 10 fractions. Fraction (0.250 g) eluted with Et_2_O (100%) showed to contain mainly inuviscolide by TLC chromatography and ^1^H NMR analysis. Further purification of this fraction was carried out on a silica-gel column (SiO_2_ 20 g) packed in CH_2_Cl_2_ and eluted first with CH_2_Cl_2_, then CH_2_Cl_2_/MeOH (99:1), CH_2_Cl_2_/MeOH (98:2), and finally MeOH. Fractions eluted with CH_2_Cl_2_/MeOH (98:2) contained inuviscolide (130 mg). Purification of a portion of this fraction was performed on a semipreparative RP-18 HPLC column eluting with CH_3_CN/H_2_O 1:1 (isocratic mode, flow rate 2 mL/min, UV detector 210 nm, R*t* 12.2 min). ^1^H and ^13^C NMR spectra (Table 1 and Table 2), MS and [α]^25^_D_ were identical to the spectroscopic data reported in literature [14,15,16].

#### Inuviscolide (**3**)

Colorless oil; R*f* 0.30 (PE/Et_2_O 3:7); [α]_D_^25^-18.9 (c, 0.05, CHCl_3_; lit. [α]_D_^25^ -18.6 (c, 0.35, CHCl_3_); UV (MeOH) λ_max_ (log ε) 204 (4.17) nm; ECD (MeOH) 211 Δε (−4.13); ^1^H and ^13^C NMR in CDCl_3_, see Table 1 and Table 2; ESI-MS: *m*/*z* 271.1307 [M+Na]^+^.

### 2.5. Dehydration of Inuviscolide to Obtain Compounds ***1*** and ***2***

An aliquot of inuviscolide (30 mg, ~0.13 mmol) was reacted with SOCl_2_ (1.2 eq) and Et_3_N (1.6 eq) in 3 mL of anhydrous CH_2_Cl_2,_ stirring overnight under Argon atmosphere. The reaction mixture was checked by TLC (PE/Et_2_O, 3:7), concentrated and purified on a semipreparative TLC to afford two main UV absorbing bands. The upper band (4.0 mg, R*f* = 0.90, PE/Et_2_O 3:7) was further purified by semipreparative RP-18 HPLC column using as eluent a mixture of CH_3_CN/H_2_O 7:3 (isocratic mode, flow rate 2 mL/min, UV detector 210 nm, R*t* 24.6 min) giving **1** (1.2 mg) as pure compound. The lower band (5.0 mg, R*f* = 0.85, PE/Et_2_O 3:7) was purified in the same HPLC condition (CH_3_CN/H_2_O 7:3, isocratic mode, flow rate 2 mL/min, UV detector 210 nm, R*t* 23.5 min) to give pure compound **2** (1.8 mg). ^1^H and ^13^C NMR, MS and [α]^25^_D_ of compounds obtained from dehydrating reaction were identical to natural centaurolide-A and -B.

### 2.6. Opening of the Lactone Ring to Obtain Alcohol ***4***

Inuviscolide (4 mg) was stirred in an aqueous solution of NaOH (5%) for 2 h. The mixture was then passed through an Amberlite XAD-2 column, first washing with water (10 mL), and then with MeOH (10 mL) to recover compound **4**. ^1^H NMR data in CD_3_OD (600 MHz): δ 5.78 (1H, brs, H-13a), 5.24 (1H, brs, H-13b), 4.95 (1H, s, H-14a), 4.85 (1H, s, H-14b), 3.56 (1H, ddd, 11.9, 9.1, 2.7 Hz, H-8), 2.61 (1H, dd, 13.8, 9.1 Hz, H-9a), 2.54 (1H, dd,13.8, 2.7 Hz, H-9b), 2.52 (1H, ddd,11.8, 9.2, 2.4 Hz, H-7), 1.92 (1H, ddd, 13.8, 1.9, 1.0 Hz, H-6a), 1.81 (1H, m, overlapped, H-1a), 1.80 (1H, m, overlapped, H-2a), 1.74 (1H, ddd, 10.2, 9.0, 9.0 Hz, H-5), 1.71 (1H, m, H-2b), 1.68 (1H, m, H-1b), 1.36 (1H, ddd, 13.8, 11.8, 11.8 Hz, H-6b), 1.12 (3H, s, H_3_-15); ^13^C NMR data: δ 178.1 (C-12), 153.6 (C-11), 150.3 (C-10), 117.6 (C-13), 112.4 (C-14), 80.5 (C-4), 78.6 (C-8), 56.2 (C-5), 52.4 (C-7), 50.2 (C-1), 46.3 (C-9), 40.7 (C-2), 33.4 (C-6), 29.4 (C-1), 23.9 (C-15); ESI-MS: *m*/*z* 265.1456 ([M-Na]^−^ negative mode).

### 2.7. Cell Viability by SRB Assay

Human cervix adenocarcinoma cells HeLa cells were obtained from ATCC-LGC Standards Repository (ATCC number CCL-2) and maintained in EMEM supplemented with 10% heat-inactivated fetal bovine serum (FBS). To test the effect of guaianolides 1–3 on cell viability, cells were seeded in regular medium with 10% FBS (1 × 10^4^ cells/well in a 96-well multi-well system). After adhesion (approximately 3 h), medium was replaced with 0.4% FBS medium and cells were exposed to increasing concentrations (1–10–25–50–100 µM) of compounds for 24 h. After treatment, medium was removed and cells were fixed with a 10% trichloroacetic acid solution through a 1 h incubation at 4 °C. To remove fixative solution, cells were washed 3 times with distilled water and further incubated with a 0.4% SRB solution for 10 min at RT in the dark. Finally, cells were washed 3 times with a 1% acetic acid solution and let to air-dry overnight. The day after, to dissolve SRB-bound protein, 10 mM of Tris-HCl (pH 10.5) were added and absorbance (by means of optical density, OD) was measured by GloMaxmultireader (Promega) equipped with a 540 nm filter. OD values from vehicle-treated cells were considered as 100% of proliferation and results were expressed as percentage (%) of the control (vehicle alone). All compounds were dissolved in DMSO and the final percentage of solvent used was less than 0.3 % per well.

### 2.8. Statistical Analysis

Data are reported as mean ± S.E.M of three independent experiments conducted in triplicates. Data were analyzed by one-way analysis of variance (ANOVA) followed by Bonferroni post hoc test. Statistical analysis was performed with GraphPad Prism 8.3 (GraphPad Software, Inc., San Diego, CA, USA).

## 3. Results

**Figure 1:**
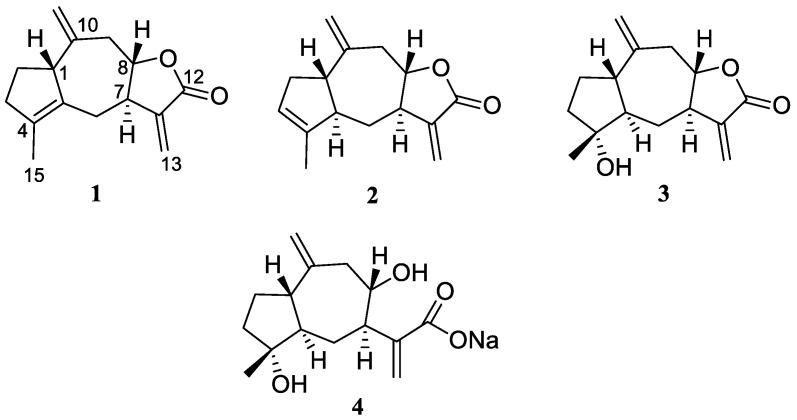
Structures of centaurolide-A (**1**), -B (**2**), inuviscolide (**3**), and alcohol derivative (**4**).

The fractionation of the CHCl_3_ extract resulted in the isolation of a series of previously reported compounds, sesquiterpenoids and polyphenols, mainly (see Supplementary Materials). The sesquiterpenoid fraction—about 10% of the extract—was mainly constituted by eudesmane compounds, α-costic acid [17,18], viscic acid [19,20], ilicic acid [21,22], 11β,13-dehydromelitensin [23,24], and 3,5,11(13)-trien-eudesma-12-oic acid [25,26], and seco-guaianolides, 1β,5β-epoxyxanthatin [27,28] and tomentosin [28,29,30]. Additionally, two new minor guaianolide metabolites, centaurolide-A (**1**) and centaurolide-B (**2**) (Figure 1), have been isolated from sesquiterpenoid fraction as described in Materials and Methods. The determination of the structure of these compounds is reported below.

Centaurolides **1** and **2** have the same molecular formula C_15_H_18_O_2_ as it was evidenced by the sodiated molecular peaks at *m*/*z* 253.1199 and 253.1197, in their positive HRESI-MS spectra, respectively. ^1^H and ^13^C NMR data of **1** and **2** (CDCl_3_, Table 1 and Table 2) indicated that both compounds are characterized by the same tricyclic sesquiterpene skeleton, exhibiting two exomethylene groups, a vinyl methyl, and a lactone functionality.

Centaurolide A (**1**) was analyzed first. The presence of a γ-lactone moiety in its structure, was suggested by the IR band at 1765 cm^−1^ and supported by the CO signal at δ 170.2 (s, CO, C-12) in the ^13^C NMR spectrum. The ^1^H NMR spectrum of **1** contains four 1H multiplets at δ 6.23 (d, *J* = 3.4 Hz, H_2_-13a), 5.59 (d, *J* = 3.1 Hz, H_2_-13b), 5.01 (br s, H_2_-14a), and 4.89 (br s, H_2_-14b) that have been assigned to two exomethylene groups, one of which α,β-conjugated with the lactone carbonyl. A series of multiplets between δ 4.30 and δ 1.80 are also present in the spectrum along with a 3H broad singlet at δ 1.70 (H_3_-15) that has been attributed to a vinyl methyl linked to a tetrasubstituted double bond. The COSY experiment shows the presence of two spin systems, the first one consistent with the sequence from H-1 (δ 3.27) to H_2_-2 (δ 2.07 and 1.87) to H_2_-3 (δ 2.39 and 2.24) in A ring, whereas the second one connects H_2_-6 (δ 2.96 and 1.81) to H-7 (δ 2.56), which is correlated to H-8 (δ 4.23), in turn coupled with H_2_-9 (δ 3.08 and 2.44), in B ring. The ^13^C NMR spectrum displays, in addition to the CO signal, fourteen resonances that have been assigned to four quaternary sp^2^ C (δ 148.1, 139.8, 134.4, and 134.1), three sp^3^ CH at δ 83.5, 53.1, and 46.5, six CH_2_, two of which are sp^2^ (δ 119.5, 112.9), and 1 CH_3_ (δ 14.2).

The analysis of the long-range correlations in the HMBC spectrum led to the definition for centaurolide A of the 8,12-guianolide skeleton with a C-4/C-5 double bond as indicated in formula **1**. Once assigned the gross structure, the relative configuration of the three stereogenic angular centers was determined by detailed analysis of *J*_HH_ coupling constant values, NOE difference, and NOESY experiments, as well as by comparison of the NMR values with those of reported *cis*- and *trans* γ-lactone fused guaianolides. In particular, the *trans* stereochemistry of C-7/C-8 junction was suggested by analysis of both multiplicity of H-8 (ddd, *J* = 10.2, 9.7 and 5.4 Hz) and H-7 (dddd, *J* = 11.5, 9.7, 6.5, 3.1 Hz), and ^13^C NMR values of C-8 (δ C 83.5) and C-7 (δ C 46.5). These data were particularly indicative of a *trans* γ-lactone arrangement as it was evident by comparison with those reported for both *cis*-fused models, as ziniolide [H-8, ddd, *J* = ~7 Hz; H-7, dddd, *J* = 11.5, ~7, 3.0, ~2 Hz] [31] and xantholide [H-8, ddd, *J* = 9.0, 7.0, 6.0 Hz; H-7, dddd, *J* = 13.0, 7.0, 3.0, 2.0 Hz] [32], and *trans*-fused models as inuviscolide [H-8, ddd, *J* = 11.0, 9.0, 6.0 Hz; H-7, ddddd, *J* = 11.0, 9.0, 6.0, 3.0, 3.0 Hz] [14,15,16]. The relative orientation of H-1 was determined by an indicative steric effect that was observed between H-1 and H-8 in the NOESY and NOE difference spectrum. Further NOE effects were detected between H-8 and H-6α, and between H-6β and both H_2_-13a and H_3_-15, in accordance with the relative configuration depicted in structure **1**. The complete NMR assignment is reported in Table 1 and Table 2.

Centaurolide B (**2**) displayed the same IR band (1769 cm-1), suggesting also for this compound a guaianolide skeleton. The ^1^H and ^13^C NMR spectra of **2** show strong similarities with those of centaurolide A (**1**). The only differences consist in the signals of two methines (C-3: δ_H_ 5.43, δ_C_ 124.2; C-5: δ_H_ 2.36, δ_C_ 54.4) replacing the allylic methylene at C-3 (δ_H_ 2.39 and 2.24, δ_C_ 37.0) and the C-5 quaternary carbon (δ_C_ 134.1) present in compound **1**, according to Δ^3^ rather than Δ^4^ double bond. The stereochemistry of the lactone junction of **2** was established to be *trans*, the same as **1**, comparing the H-7 and H-8 multiplicity pattern, and the carbon values of C-7, C-8, and C-9 with those of **1** and literature models [16,33]. A detailed analysis of a series of NOE difference and NOESY experiments led us to assign the *trans* relative configuration also for the C-1/C-5 ring junction. In fact, steric effects were observed between H-1 (δ 2.52, m) and H-8 (δ 4.37, ddd, *J* = 11.4, 9.4 and 4.6 Hz) as well as between H-5 (δ 2.36, m) and H-7 (δ 2.70, dddd, *J* = 10.3, 9.4, 6.4, 3.2 Hz) according to the proposed structure **2**. The ring junction stereochemistry of **2** is the same as the closely related inuviscolide (**3**) (Table 1), a well-known 8,12-guaianolide firstly reported from the medicinal plant *Dittrichia viscosa* (L.) Greuter [14,15,16] and later isolated from other *Inula* species [34]. In particular, from a structural point of view, compounds **1** and **2** are the endo dehydration derivatives of **3** from which they could biogenetically derive. Inuviscolide (**3**) (Figure 1) was not detected in the CHCl_3_ extract of *C. omphalotricha*. Its structural relationship with new centaurolides **1** and **2** was chemically confirmed by dehydration of an authentic sample of **3** isolated from an expressly collected *D. viscosa* specimen (see Experimental Part). The two main dehydration products obtained from **3** showed spectroscopic data (^1^H and ^13^C NMR, ESIMS) and optical properties ([α]^25^_D_values and CD profiles) the same as centaurolides A (**1**) and B (**2**) (see Experimental Part), thus confirming the proposed structures and the relationship with **3**, including the same absolute configuration.

It should be noted, however, that the absolute configuration of inuviscolide (**3**) has not been reported in the literature to date and, consequently, it was not assayed for centaurolides A (**1**) and B (**2**). Thus, with the aim at assessing the absolute configuration of all three compounds **1**–**3**, we tried to apply the Mosher’s method to the alcohol **4** (Figure 1) that was obtained by opening of the lactone ring from compound **3** [35], which was available in a larger amount with respect to **1** and **2**. Unfortunately, compound **4** resulted to be very unstable under Mosher esterification conditions, undergoing a rapid cyclization to form the starting lactone ring and any attempt to obtain the MTPA esters failed. Thus, the absolute configuration of **1–3** remains undetermined.

**Figure 2:**
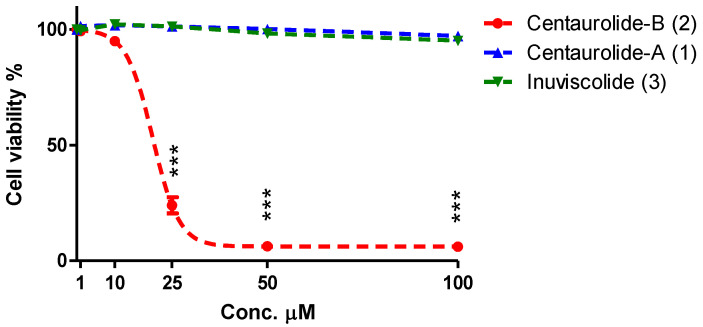
Effect of centaurolide-A (**1**), -B (**2**) and inuviscolide (**3**) on cell viability in human cervix adenocarcinoma cells (HeLa cells). Hela cells were treated with various concentrations (1, 10, 25, 50, 100 μM) of centaurolide-A (**1**), centaurolide-B (**2**), and inuviscolide (**3**), for 24 h and then cell viability was determined by SRB assay. Data has been plotted with non-linear regression. Different data points represent mean ± standard error (SE) of three independent experiments (n = 3); *** *p* < 0.001 compared to the corresponding vehicle. Statistical analysis was carried out using one-way ANOVA followed by Bonferroni as post hoc test.

In order to assess the contribution of centaurolides A and B to the inhibitory activity of *C. omphalotricha* CHCl_3_ extract on the viability of human cervical cancer cells, pure compounds **1** and **2** were assayed, along with inuviscolide (**3**), on human cervix adenocarcinoma HeLa cells. A five-point concentration-response was tested for each compound, and after 24 h treatment, activity was measured by SRB assay. Interestingly, while centaurolide-A (**1**) and inuviscolide (**3**) were not active up to the highest concentration tested (100µM), centaurolide-B (**2**) showed a concentration-dependent inhibitory effect on cell viability (IC50 18 μM) (Figure 2).

## 4. Discussion and Conclusions

Plants belonging to the genus *Centaurea* are characterized by the ability to produce flavonoids [36] and sesquiterpene lactones [5], which are important chemotaxonomic markers. The sesquiterpene profile of *Centaurea* species comprises mainly germacrane, elemane, eudesmane, and guaiane skeletons [5]. From a structural point of view, the majority of these sesquiterpenoids in *Centaurea* have an α-oxygenated function at C-6 that, normally, is involved in the formation of the C-6/C-12 γ-lactone or, in some cases, is present as a free hydroxyl group. Differently from the chemistry of other genera of the Asteraceae family, the oxygenated function at C-8 is almost always α-orientated and, if present, is esterified [5].

According to literature data reported for *Centaurea* genus, the CHCl_3_ extract of *C. omphalotricha* from Bousaada, northeast Algeria, showed a chemical pattern characterized by sesquiterpenoid and polyphenolic compounds. In particular, eudesmane-based compounds as α-costic, viscic, and ilicic acids were the main components of the sesquiterpenoidic fraction, whereas the seco-guaianolides tomentosin and 1β,5β-epoxyxanthatin along with new 12,8-guaianolides centaurolides A and B were found as minor components.

This sesquiterpenoidic pool composition distinguishes the chemistry of the sample we studied from that described for *C. omphalotricha* collected near Bechar in the southwest of the country, that is reported to be characterized by a series of 12,6-guaianolide sesquiterpenoids [13].

The finding of centaurolides **1** and **2** in *C. omphalotricha*, even if in low amount, represents the first report of 12,8 guaianolide skeleton in *Centaurea* genus. The structures of new compounds **1** and **2** resemble that of inuviscolide (**3**) being centaurolides its endo dehydration derivatives, as confirmed by dehydration of **3**. However, inuviscolide (**3**), which has been reported mainly from species of *Dittrichia* (=*Inula*) genus, has not been detected in our sample of *C. omphalotricha*. Thus, a possible derivation of compounds **1** and **2** by workup conversion of **3** seems improbable. In addition, compound **3** appeared to be very stable under all chromatographic conditions used for its purification and dehydration products of **3** were not observed to be formed.

Biological studies have revealed significant pharmacological properties associated to several *Centaurea* extracts, explaining the long-term use of the genus in traditional medicine of distinct countries [3]. It has been evidenced that the main responsible of the bioactivity are sesquiterpene lactones and, in particular, guaianolides including 12,6- and 12,8-guaiane lactones as well as rearranged pseudoguaiane lactones [5,37,38]. Despite the low distribution in nature of 12,8-guaianolides with respect to compounds with a 12,6-olide framework, these members of the guaianolide family exhibit the broadest spectrum of biological activity including anticancer, anti-inflammatory, antimicrobial properties [37,38]. However, within the 12,8-guaianolides isolated in this work, only centaurolide B (**2**) showed a moderate antiproliferative effects against HeLa cell lines. 

In summary, this study provides new insights on the chemistry of poorly studied Algerian *C. omphalotricha*. Sesquiterpenoids with 12.8-guaianolide skeleton have been found for the first time in *Centaurea* genus.

## Figures and Tables

**Table 1:** ^1^H NMR data ^a–c^ in CDCl_3_ for centaurolide A (**1**) and B (**2**), and inuviscolide (**3**) ^d^.

C	1	2	3
	*δ*_H_ (*J*, Hz)	*δ*_H_ (*J*, Hz)	*δ*_H_ (*J*, Hz)
1	3.27 (m, *W*_1/2_ 18)	2.52 (m)	2.17 (dd, 8.6, 8.2)
2a 2b	2.07 (m) 1.87 (m)	2.51 (m) 2.31 (m)	1.97 (m) 1.72 (m)
3a 3b	2.39 (br dd, 7.4, 7.4) 2.24 (m)	5.43 (br s)	1.83 (m) 1.71 (m)
4			
5		2.36 (m)	1.67 (ddd, 11.8, 11.6, 3.8)
6a 6b	2.96 (dd, 14.2, 3.1) 1.81 (dd, 14.2, 11.5)	2.47 (ddd, 13.0, 3.2, 3.1) 1.15 (ddd, 13.0, 13.0, 10.3)	2.29 (ddd, 13.2, 6.2, 3.8) 1.23 (ddd, overlapped)
7	2.56 (dddd, 11.5, 9.7, 6.5, 3.1)	2.70 (dddd, 10.3, 9.4, 6.4, 3.2)	2.66 (dddd, 10.6, 9.5, 6.2, 3.1)
8	4.23 (ddd, 10.2, 9.7, 5.4)	4.37 (ddd, 11.4, 9.4, 4.6)	4.31 (ddd, 10.7, 9.5, 6.1)
9a 9b	3.08 (b dd, 13.8, 5.4) 2.44 (dd, 13.8, 10.2)	3.29 (ddd, 16.3, 4.6, 2.2) 2.66 (dd, 16.3, 11.4)	3.21 (dd, 15.4, 6.1) 2.56 (dd, 15.4, 10.7)
10			
11			
12			
13a 13b	6.23 (d, 3.4) 5.59 (d, 3.1)	6.25 (d, 3.4) 5.63 (d, 3.0)	6.23 (d, 3.4) 5.55 (d, 3.1)
14a	5.01 (br s)	5.06 (br s) 4.99 (br s)	5.10 (br s) 4.97 (br s)
14b	4.89 (br s)		
15	1.70 (br s)	1.70 (br s)	1.20 (s)

^a^ Spectra recorded on 600 and 400 MHz instruments; ^b^ Assignments aided by COSY, HSQC edited, and HMBC (*J* = 7 Hz) experiments; ^c^ Coupling constant determined by homodecoupling experiments; ^d^ NMR data acquired on an authentic sample isolated in this study.

**Table 2:** ^13^C NMR data ^a,b^ in CDCl_3_ for centaurolide A (**1**) and B (**2**), and inuviscolide (**3**) ^c^.

C	1	2	3
	*δ*_C_ type	*δ*_C_ type	*δ*_C_ type
1	53.1, CH	51.0, CH	47.0, CH
2	29.8, CH_2_	34.5, CH_2_	26.3, CH_2_
3	37.0, CH_2_	124.2, CH	41.3 CH_2_
4	134.4, C	140.7, C	80.5, C
5	134.1, C	54.4, CH	59.2, CH
6	28.1, CH_2_	32.8, CH_2_	30.0, CH_2_
7	46.5, CH	47.9, CH	45.4, CH
8	83.5, CH	81.2, CH	82.4, CH
9	40.4, CH_2_	40.5, CH_2_	40.7, CH_2_
10	148.1, C	145.0, C	146.6, C
11	139.8, C	139.8, C	139.6, C
12	170.2, C	171.4, C	170.1, C
13	119.5, CH_2_	119.8, CH_2_	120.5, CH_2_
14	112.9, CH_2_	111.7, CH_2_	111.8, CH_2_
15	14.2, CH_3_	14.9, CH_3_	24.2, CH_3_

^a^ Spectra recorded on 600 and 400 MHz instruments; ^b^ Assignments aided by COSY, HSQC edited, and HMBC (*J* = 7 Hz) experiments; ^c^ NMR data acquired on an authentic sample isolated in this study.

## Data Availability

Not applicable.

## Supplementary Materials

The followings are available online at https://www.mdpi.com/article/10.3390/biom11071053/s1: Detailed experimental part description of known compounds; 1D and 2D NMR spectra for compounds **1**–**4**; HRESI-MS of new compounds **1** and **2**.
