# Supplementary material for: Sesquiterpene Lactones with the 12,8-Guaianolide Skeleton from Algerian Centaurea omphalotricha
Source: Biomolecules. 2021 Jul 18;11(7):1053. doi: 10.3390/biom11071053 (PMC8301927; doi:10.3390/biom11071053)
Supplement: Supplementary file 1 [file biomolecules-11-01053-s001.zip › biomolecules-1281232-supplementary.pdf]

**Supplementary Material**

# Sesquiterpene lactones with the 12,8- guaianolide skeleton from Algerian *Centaurea omphalotricha*

Sara Kebbi[1,2] Maria Letizia Ciavatta[1,*] Ali Mokhtar Mahmoud[1], Marianna Carbone[1], Alessia Ligresti[1], Ramdane Seghiri[2], and Margherita Gavagnin[1]

[1]*Consiglio Nazionale delle Ricerche (CNR), Istituto di Chimica Biomolecolare (ICB), Via Campi Flegrei, 34 -80078- Pozzuoli, Napoli, Italy*

[2] *Unit of Valorization of Natural Resources, Bioactive Molecules and Physicochemical and Biological Analyzes (VARENBIOMOL), Department of Chemistry, Faculty of Exact Sciences, University of Mentouri Brothers Constantine 1, 25000 Constantine, Algérie*

***Corresponding author**

Email*:* lciavatta@icb.cnr.it; Phone +39 081 867 52 43 Fax +39 081 804 17 70

*S1.* Description of isolation of known compounds from *C.omphalotricha*

*S2.* Chemical structures of known compounds isolated from *C.omphalotricha*

*S3.* $^{1}$H NMR spectrum of centaurolide A (**1**) (600 MHz, $CDCl_3$)

*S4.* $^{13}$C NMR spectrum of centaurolide A (**1**) (100 MHz, $CDCl_3$)

*S5.* COSY spectrum of centaurolide A (**1**) (600 MHz, $CDCl_3$)

*S6.* ed-HSQC spectrum of centaurolide A (**1**) (600 MHz, $CDCl_3$)

*S7.* HMBC spectrum of centaurolide A (**1**) (600 MHz, $CDCl_3$)

*S8.* NOESY spectrum of centaurolide A (**1**) (600 MHz, $CDCl_3$)

*S9.* HR ESIMS spectrum of centaurolide A (**1**)

*S10.* $^{1}$H NMR spectrum of centaurolide B (**2**) (600 MHz, $CDCl_3$)

*S11.* $^{13}$C NMR spectrum of centaurolide B (**2**) (100 MHz, $CDCl_3$)

*S12.* COSY spectrum of centaurolide B (**2**) (400 MHz, $CDCl_3$)

*S13.* HSQC spectrum of centaurolide B (**2**) (400 MHz, $CDCl_3$)

*S14.* HMBC spectrum of centaurolide B (**2**) (600 MHz, $CDCl_3$)

*S15.* NOESY spectrum of centaurolide B (**2**) (600 MHz, $CDCl_3$)

*S16.* HR ESIMS spectrum of centaurolide B (**2**)

*S17.* $^{1}$H NMR spectrum of inuviscolide (**3**) (600 MHz, $CDCl_3$)

*S18.* $^{13}$C NMR spectrum of inuviscolide (**3**) (150 MHz, $CDCl_3$)

*S19.* COSY spectrum of inuviscolide (**3**) (600 MHz, $CDCl_3$)

*S20.* ed-HSQC spectrum of inuviscolide (**3**) (600 MHz, $CDCl_3$)

*S21.* HMBC spectrum of inuviscolide (**3**) (600 MHz, $CDCl_3$)

*S22.* NOESY spectrum of inuviscolide (**3**) (600 MHz, $CDCl_3$)

*S23.* $^{1}$H NMR spectrum of dehydration derivative of inuviscolide (600 MHz, $CDCl_3$)

*S24.* $^{13}$C NMR spectrum of dehydration derivative of inuviscolide (600 MHz, $CDCl_3$)

*S25.* $^{1}$H NMR spectrum of dehydration derivative of inuviscolide (600 MHz, $CDCl_3$)

*S26.* $^{13}$C NMR spectrum of dehydration derivative of inuviscolide (600 MHz, $CDCl_3$)

*S27.* $^{1}$H NMR spectrum of alcohol (**4**) (600 MHz, $CDCl_3$)

*S28.* $^{13}$C NMR spectrum of alcohol (**4**) (150 MHz, $CDCl_3$)

*S29.* COSY spectrum of alcohol (**4**) (600 MHz, $CDCl_3$)

*S30.* ed-HSQC spectrum of alcohol (**4**) (600 MHz, $CDCl_3$)

*S31.* HMBC spectrum of alcohol (**4**) (600 MHz, $CDCl_3$)

*S32.* NOESY spectrum of alcohol (**4**) (600 MHz, $CDCl_3$)

# *S1*

A portion (3.3 g) of the $CHCl_3$ extract of *C. omphalotricha* was first fractionated by $SiO_2$-gel column chromatography by eluting with a gradient of $CH_2Cl_2$ in petroleum ether (PE), and subsequently with a gradient of acetone in $CH_2Cl_2$ to obtain 43 fractions (C1-C43). Nine selected fractions (C13, C16, C19, C-24, C27, C29, C30, C34, C37) were taken into consideration after TLC chromatography analysis and preliminary $^1$H-NMR inspection. Fraction C13 (13.0 mg) was loaded onto a semipreparative TLC and developed with $CHCl_3$/Acetone, 8:2 to yield a UV band at R*f* 0.5 corresponding to vanillin [1,2]. A portion (50 mg) of fraction C16 (86 mg) was loaded onto a $AgNO_3$ silicagel column using as eluent petroleum ether and increasing amount of $Et_2O$. Subfractions eluted with petroleum ether/$Et_2O$ 6:4 and 1:1 contained 2 mg of pure α-costic acid. A small part (20 mg) of fraction C19 (65.5 mg) was purified by semipreparative TLC with petroleum ether/$Et_2O$ 1:1 to yield methyl-4-hydroxy benzoate (2.0 mg) [3,4]. A portion of C24 (32.3 mg) was also purified by semipreparative TLC in *n*-hexane/AcOEt 1:1 to give two UV compounds, tomentosin (2.0 mg) at R*f* 0.6 and 1β,5β-epoxyxanthatin at R*f* 0.5. Fraction C27 (94.8 mg) was purified by silicagel column using as eluent system petroleum ether with increasing amount of Et2O to give 12 subfractions. Subfraction C27-7 (27.7 mg) eluted with petroleum ether/$Et_2O$ 4:6 contained scopoletin (5.0 mg) [5,6] that was further purified by recrystallization in MeOH. Fraction C29 (200 mg) was loaded onto a silicagel column packed in PE and eluted initially with an increasing gradient of $Et_2O$ in PE (10:0, 9:1, 8:2, 7:3, 6:4, 5:5, 4:6, 3:7, 2:8, 1:9, 0:10), then with $CHCl_3$, and finally with MeOH affording 13 subfractions C29-1 – C29-13. (for the new compounds see main text). Subfraction C29-6 (12.0 mg) was loaded onto a semipreparative TLC in petroleum ether/$Et_2O$ 1:1 showing a UV band at Rf 0.45 that was identified as 3,5,11(13)-trien-eudesma-12-oic acid. Subfraction C29-10 (10.2 mg) was purified by semipreparative TLC ($CHCl_3$/MeOH, 9.5:0.5) to give 2.7 mg of 11β,13-dehydromelitensin. A portion (10.0 mg) of fraction C30 (54.4 mg) was purified by semipreparative TLC ($CHCl_3$/$Et_2O$, 1:1) to obtain 1.0 mg of cirsimaritin [7,8]. Half portion (130 mg) of fraction C34 (260.0 mg) was purified by silicagel column in a gradient of Et2O in petroleum ether to give 11 subfraction C34-1-C34-11. Subfraction C34-7 (20.0 mg) was further purified on a 10% $AgNO_3$ silicagel pipette Pasteur using a gradient of petroleum ether in $CHCl_3$ to yield 5.3 mg of viscic acid. A portion (150.0 mg) of fraction C37 (203.0 mg) was fractionated by silicagel column using first a gradient of $Et_2O$ in petroleum ether, then CHCl3 and MeOH to give 15 subfractions. Subfraction C37-7 (28.7 mg) was recrystallized in MeOH to get 5.0 mg of ilicic acid.


1. Tiemann, F.; Haarmann, W. Ueber das Coniferin und seine Umwandlung in das aromatische Princip der Vanille. *Berichte der Dtsch. Chem. Gesellschaft* **1874**, *7*, 608–623, doi:10.1002/cber.187400701193.
2. Mukonyi, K.W.; Ndiege, I.O. 2-Hydroxy-4-methoxybenzaldehyde: Aromatic taste modifying compound from *Mondia whytei* Skeels. *Bull. Chem. Soc. Ethiop.* **2001**, *15*, 137-141, doi:10.4314/bcse.v15i2.20959.
3. Jong, T.-T.; Jean, M.-Y. Constituents of *Houttuyniae cordata* and the Crystal Structure of Vomifoliol. *J. Chinese Chem. Soc.* **1993**, *40*, 399–402, doi:10.1002/jccs.199300062.
4. Yoshioka, T.; Inokuchi, T.; Fujioka, S.; Kimura, Y. Phenolic compounds and flavonoids as plant growth regulators from fruit and leaf of *Vitex rotundifolia*. *Zeitschrift fur Naturforsch. - Sect. C J. Biosci.* **2004**, 509-514, doi:10.1515/znc-2004-7-810.
5. Eijkman, J.F. Sur les principes vénéneux de la *Scopolia japonica*. *Recl. des Trav. Chim. des Pays-Bas* **1884**, *3*, 169–181, doi:10.1002/recl.18840030602.
6. Vasconcelos, J.M.J.; Silva, A.M.S.; Cavaleiro, J.A.S. Chromones and flavanones from *Artemisia campestris* subsp. *maritima*. *Phytochemistry* **1998**, *49*, 1421–1424, doi:10.1016/S0031-9422(98)00180-0.
7. Brieskorn, C.H.; Biechele, W. 6-Methoxygenkwanin - ein weiteres flavon aus labiaten. *Tetrahedron Lett.* **1969**, *10*, 2603–2605, doi:10.1016/S0040-4039(01)88222-8.
8. Wang, R.-F.; Yang, X.-W.; Ma, C.-M.; Liu, H.-Y.; Shang, M.-Y.; Zhang, Q.-Y.; Cai, S.-Q.; Park, J.-H. Trollioside, a new compound from the flowers of *Trollius chinensis*. *J. Asian Nat. Prod. Res.* **2004**, *6*, 139–144, doi:10.1080/1028602031000147393.

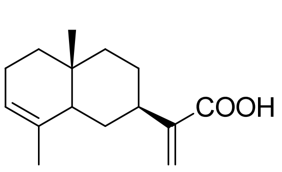

α-costic acid

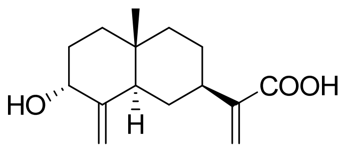

viscic acid

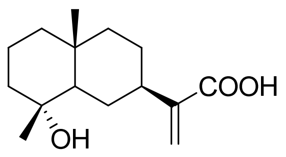

liicic acid

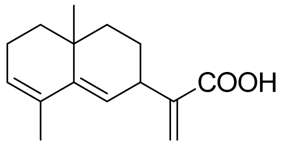

3,5,11-trien-eudesman-12-oic acid

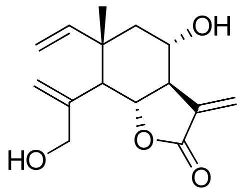

11β,13-dehydromelitensin

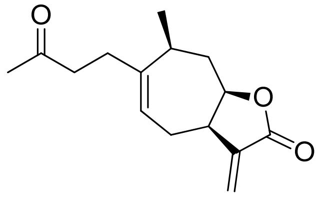

tomentosin

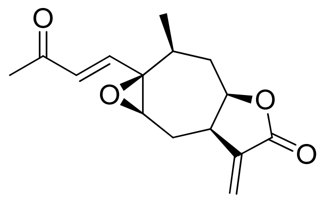

1β,5β-epoxyxanthatin

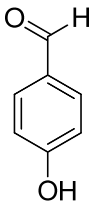

vanillin

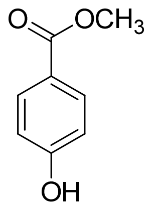

4-OH-Me-benzoate

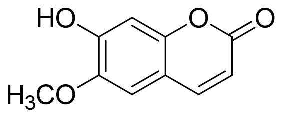

scopoletin

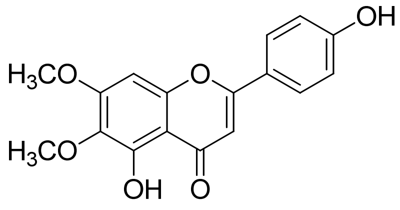

cirsimaritin

*S2*. Chemical structures of known compounds isolated from *C.omphalotricha*

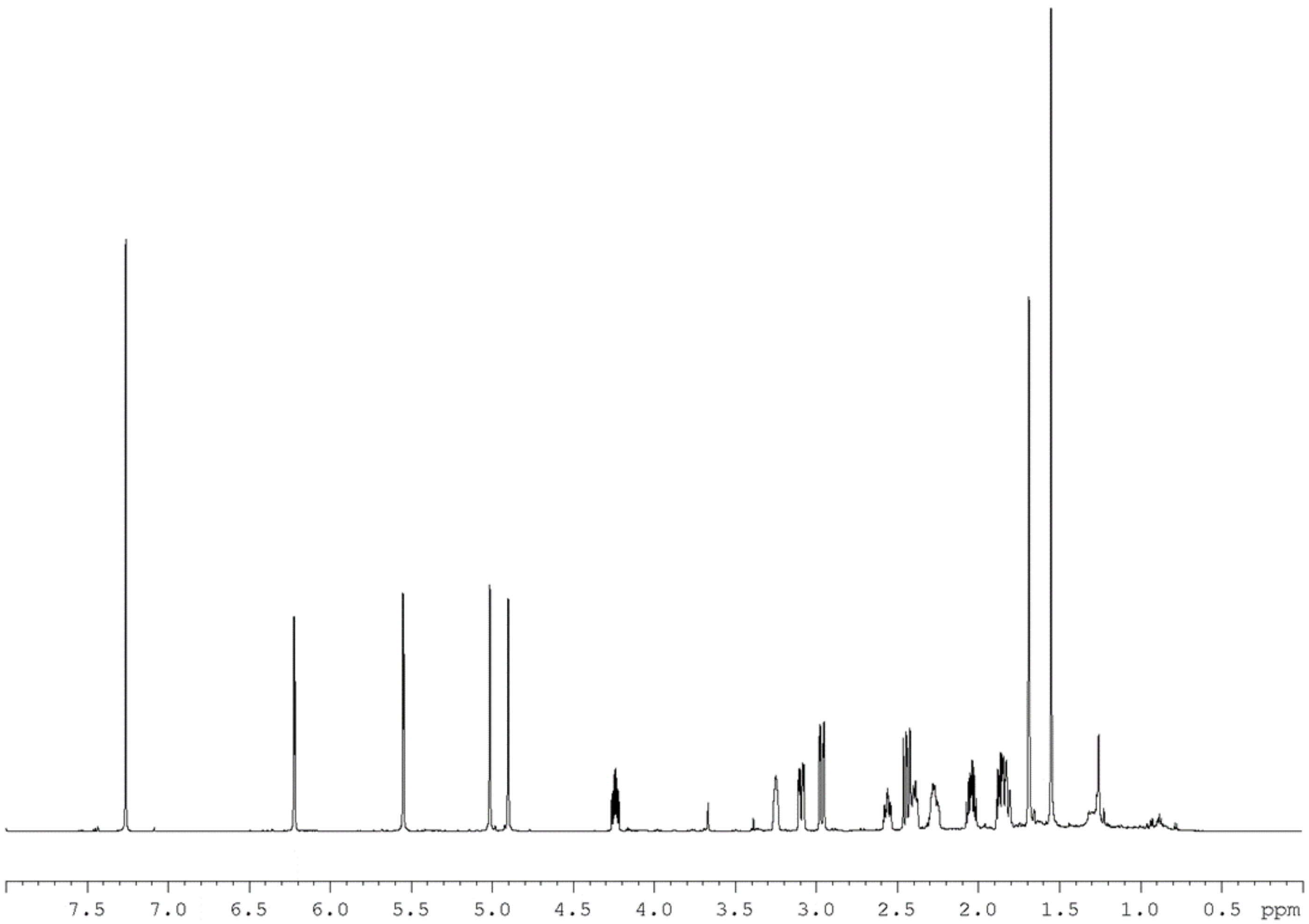


*S3*. $^{1}$H NMR spectrum of centaurolide A (**1**) (600 MHz, $CDCl_3$)

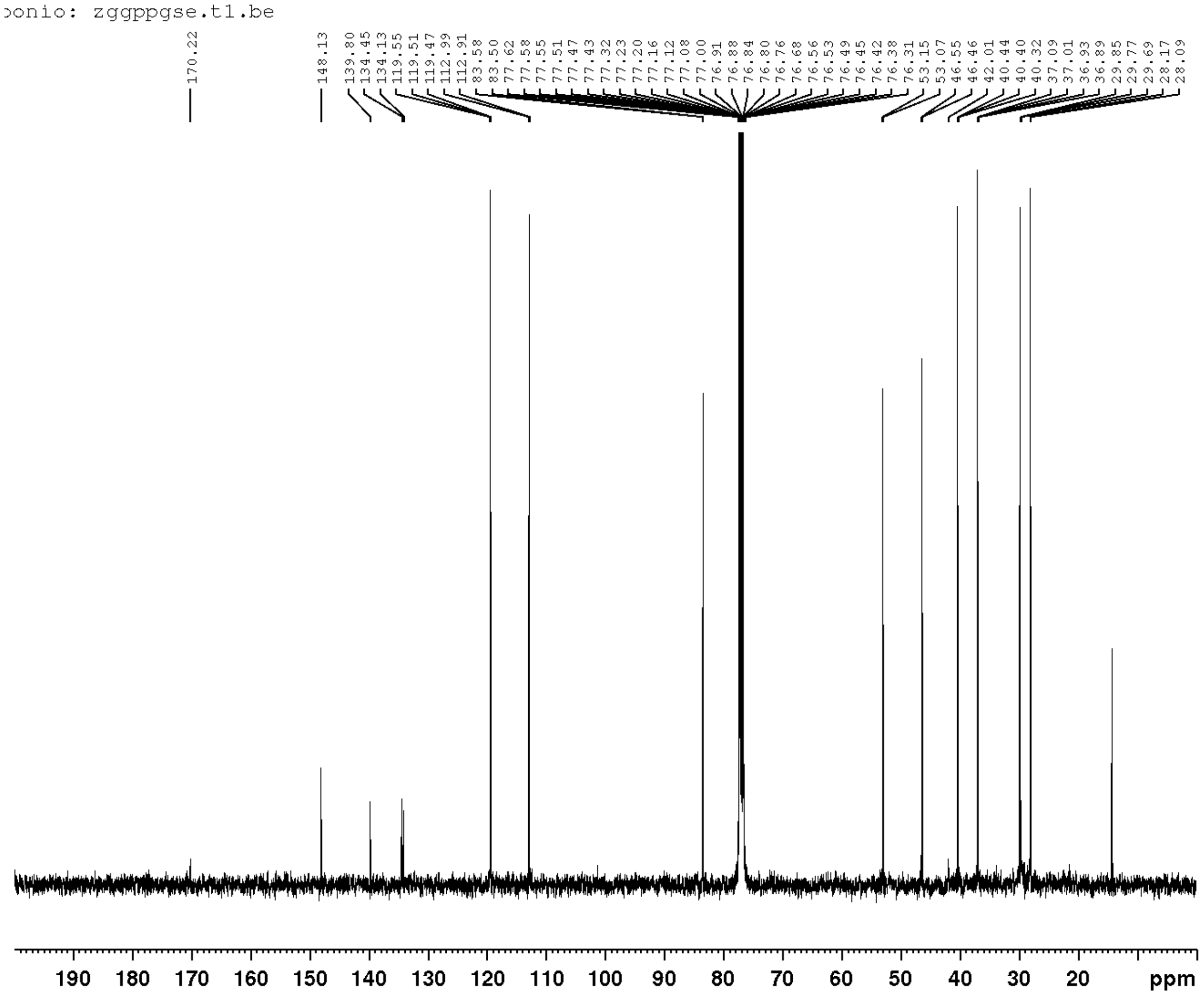


*S4*. $^{13}$C NMR spectrum of centaurolide A (**1**) (100 MHz, $CDCl_3$)

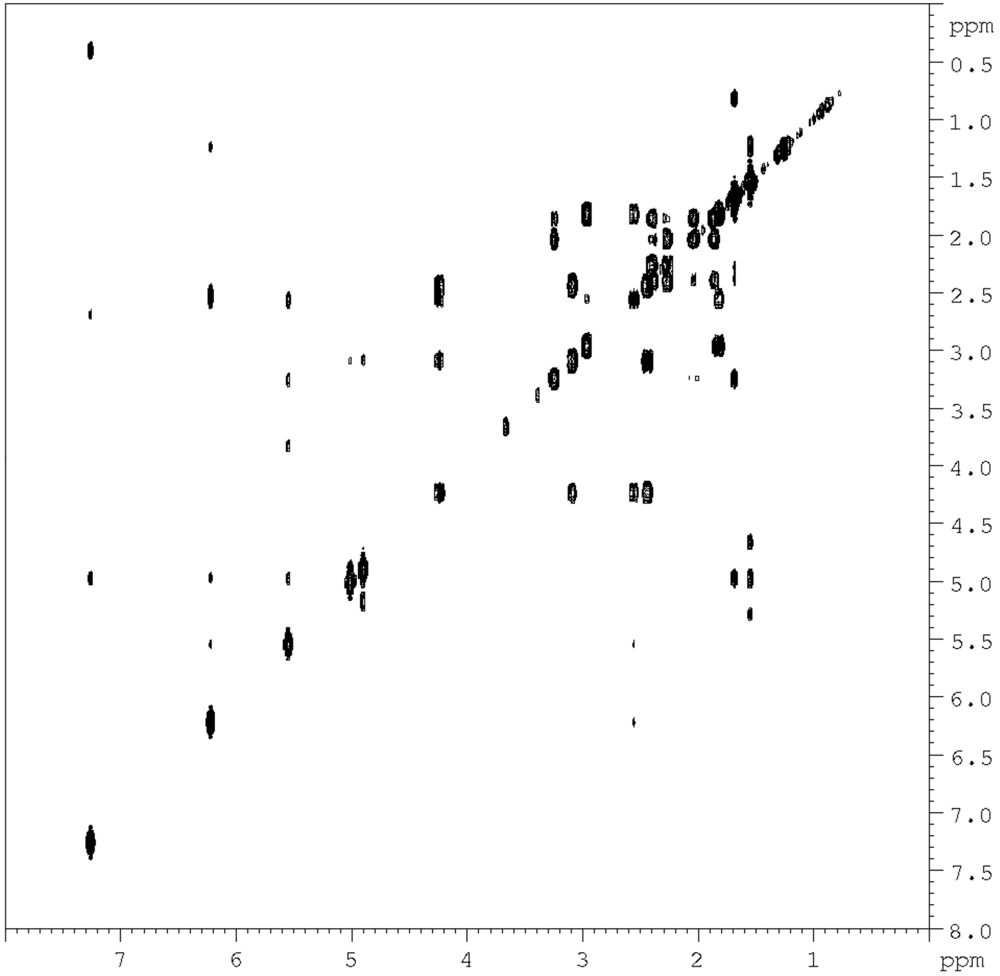


*S5*. COSY spectrum of centaurolide A (**1**) (600 MHz, $CDCl_3$)

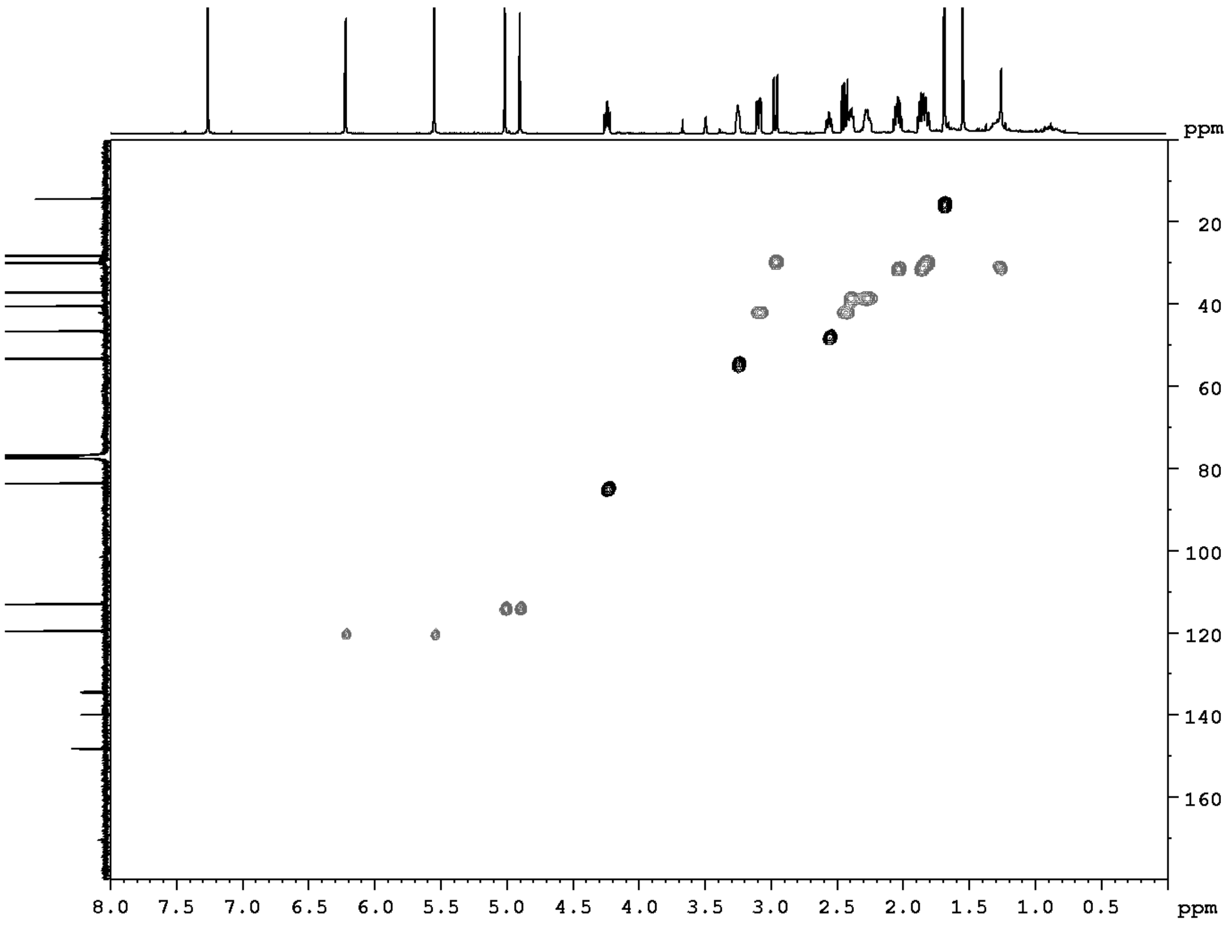


*S6.* ed-HSQC spectrum of centaurolide A (**1**) (600 MHz, $CDCl_3$)

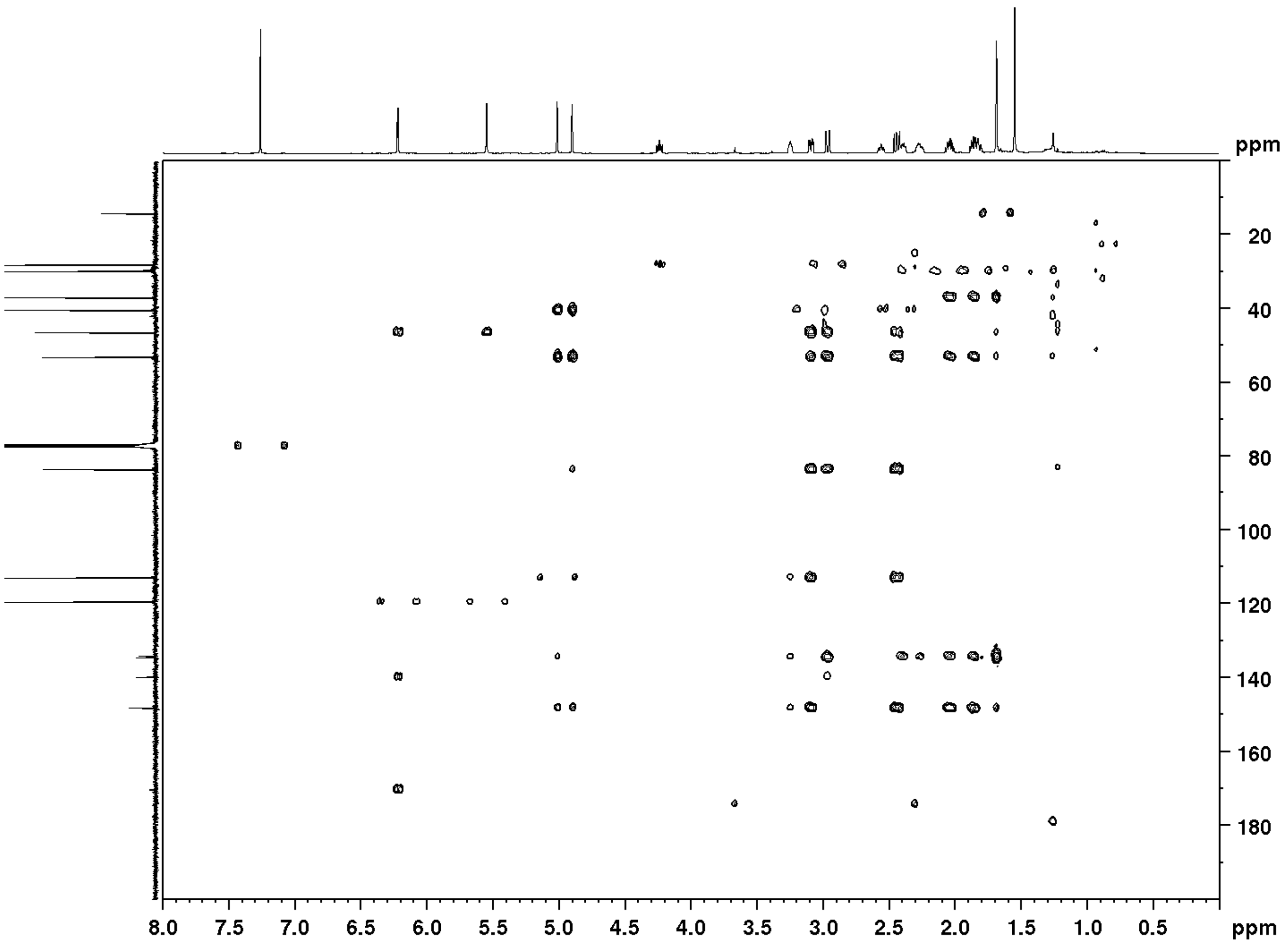


*S7*. HMBC spectrum of centaurolide A (**1**) (600 MHz, $CDCl_3$)

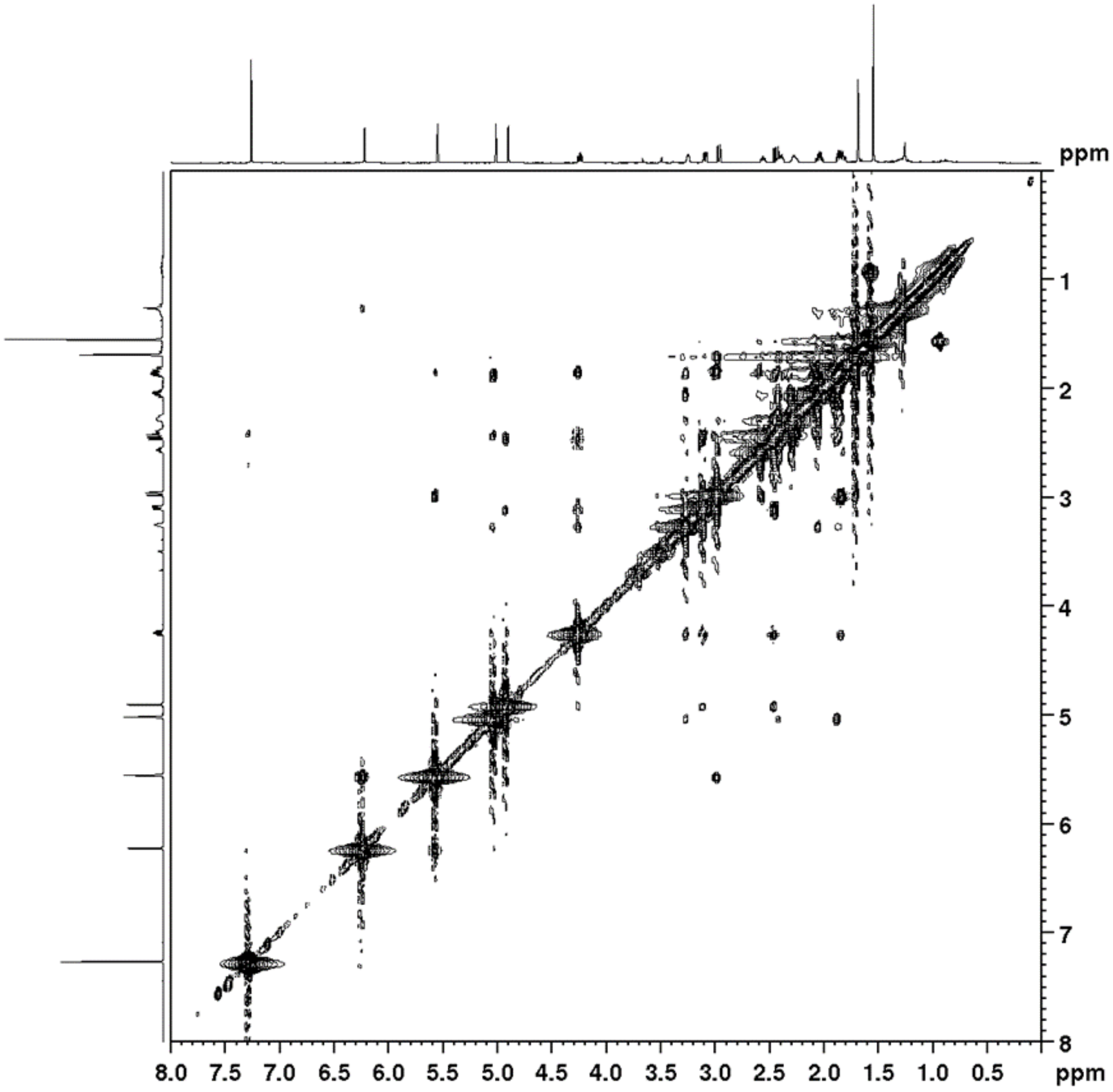


*S8*. NOESY spectrum of centaurolide A (**1**) (600 MHz, CDCl3.

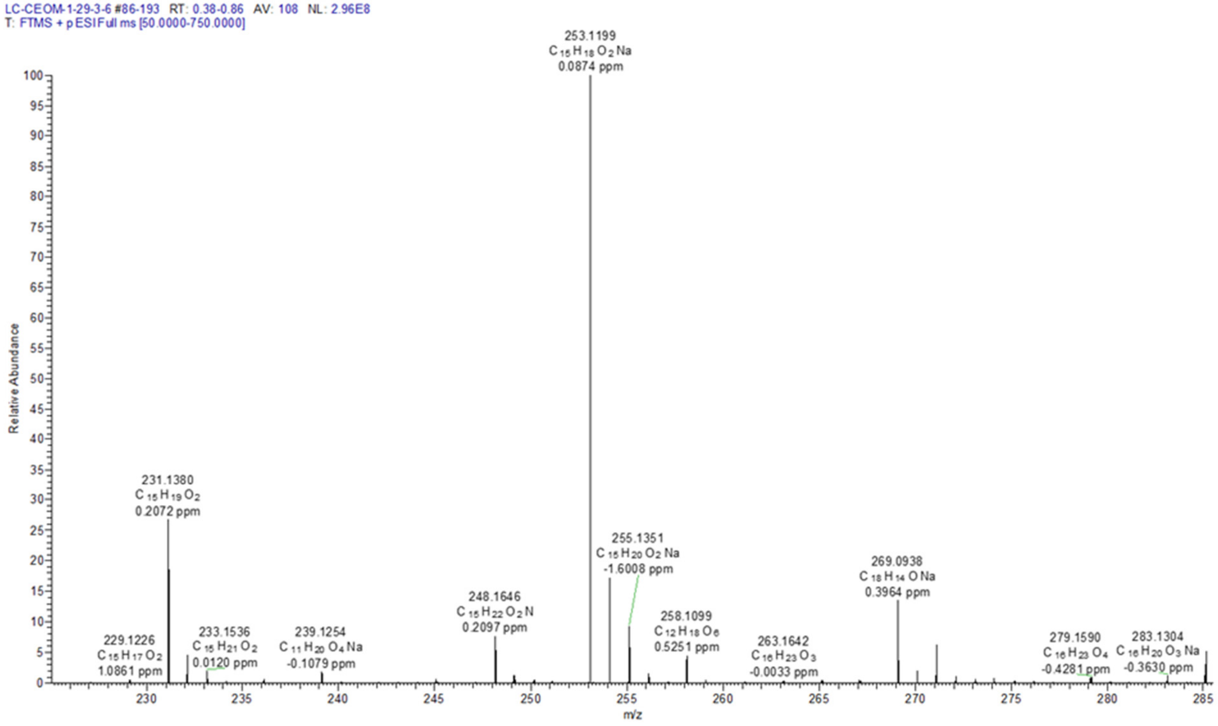


*S9*. HR ESIMS spectrum of centaurolide A (**1**)

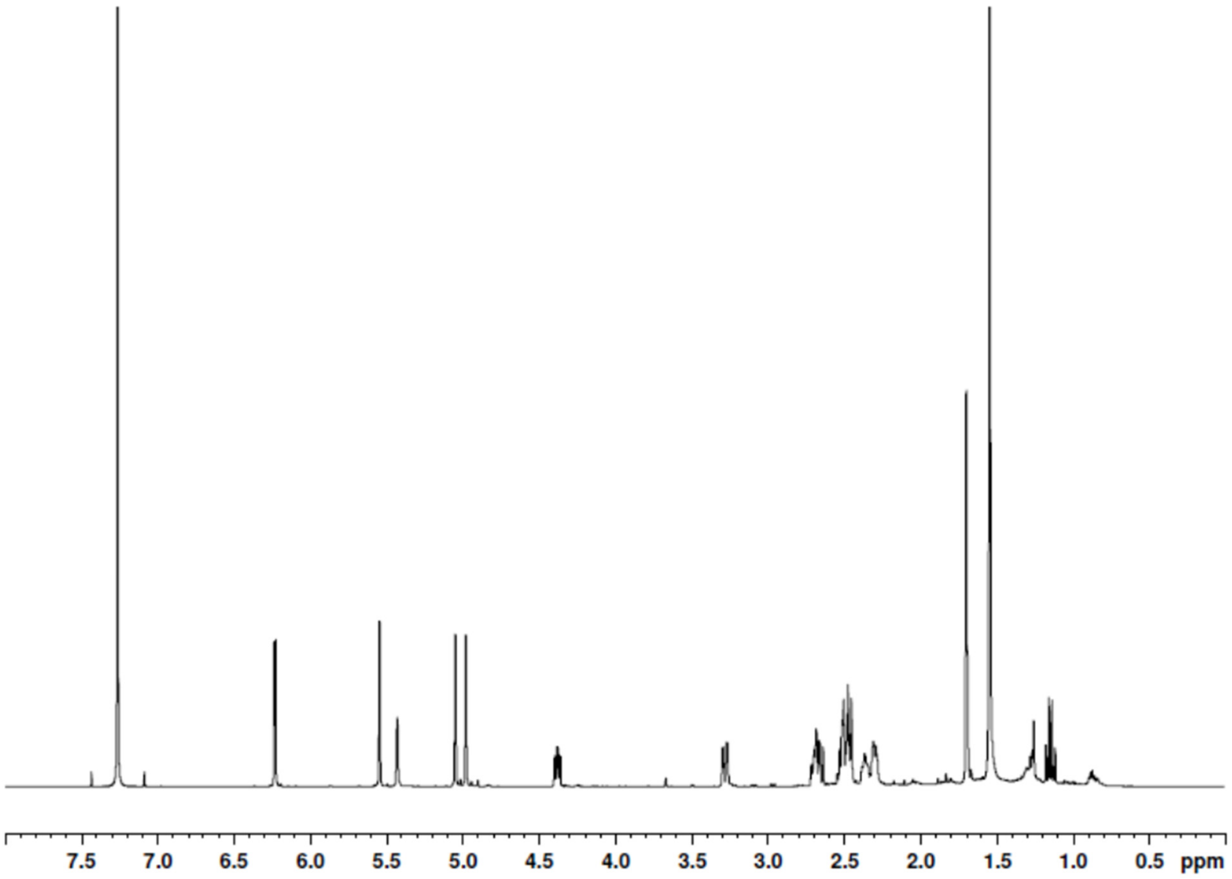


*S10.* $^{1}$H NMR spectrum of centaurolide B (**2**) (600 MHz, $CDCl_3$)

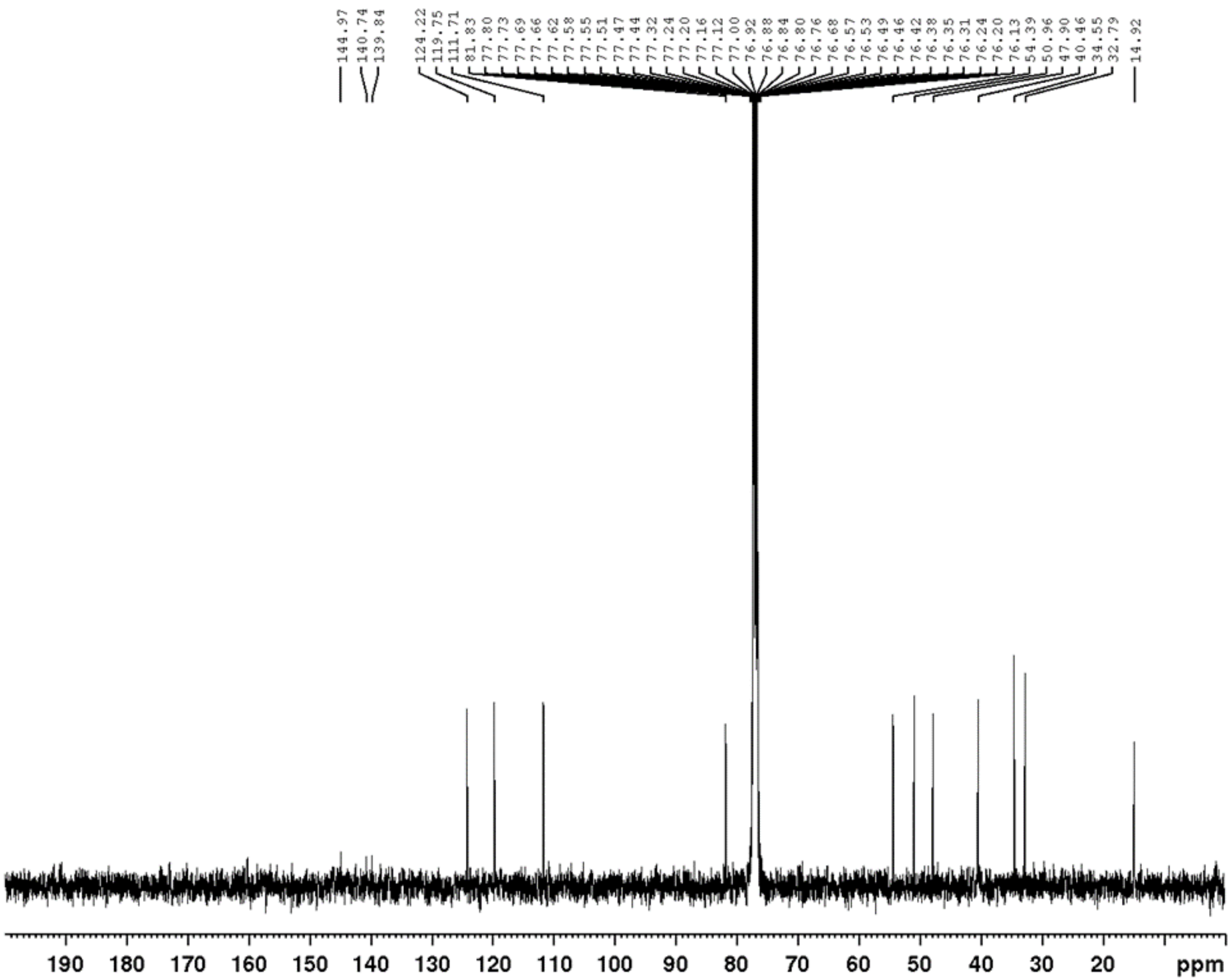


*S11.* $^{13}C$ NMR spectrum of centaurolide B (**2**) (100 MHz, $CDCl_3$)

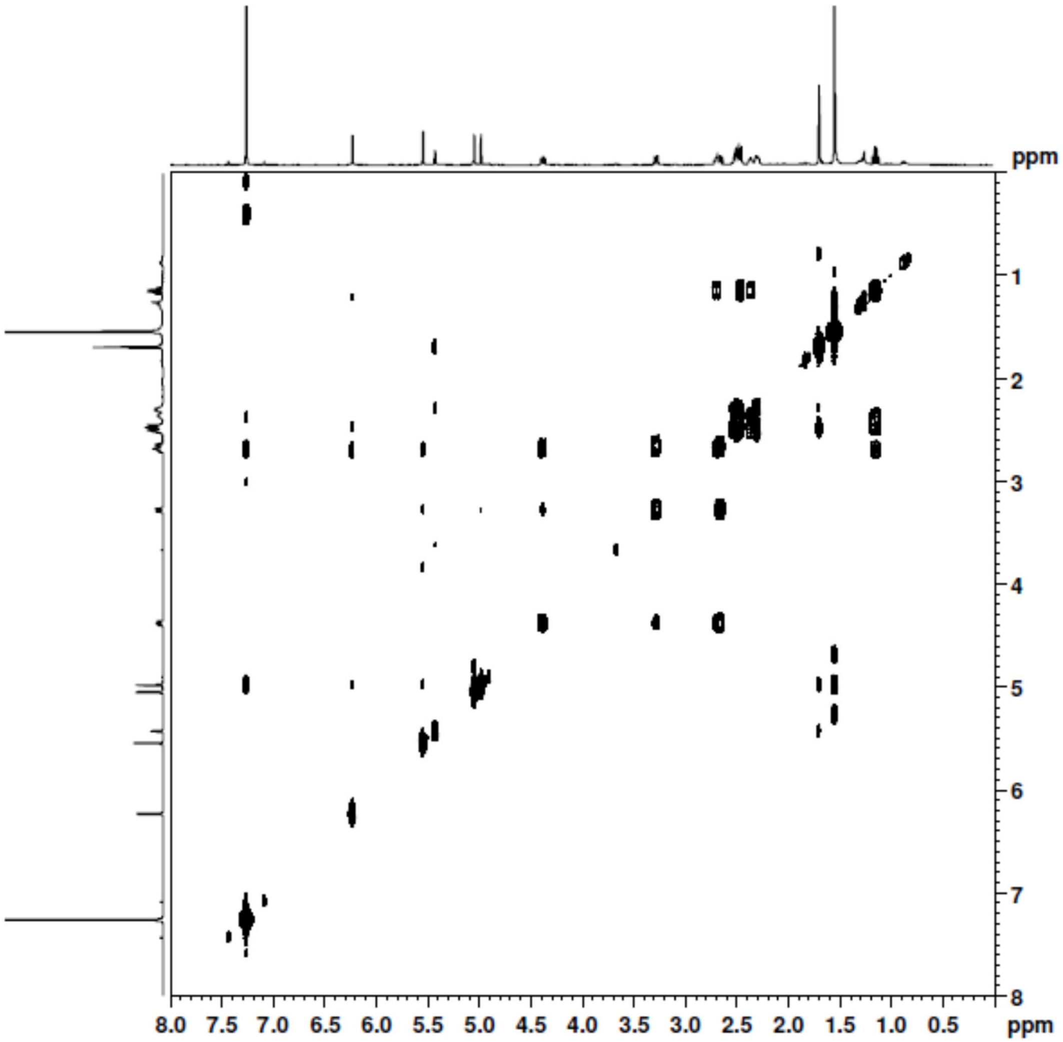


*S12.* COSY spectrum of centaurolide B (**2**) (400 MHz, CDCl3)

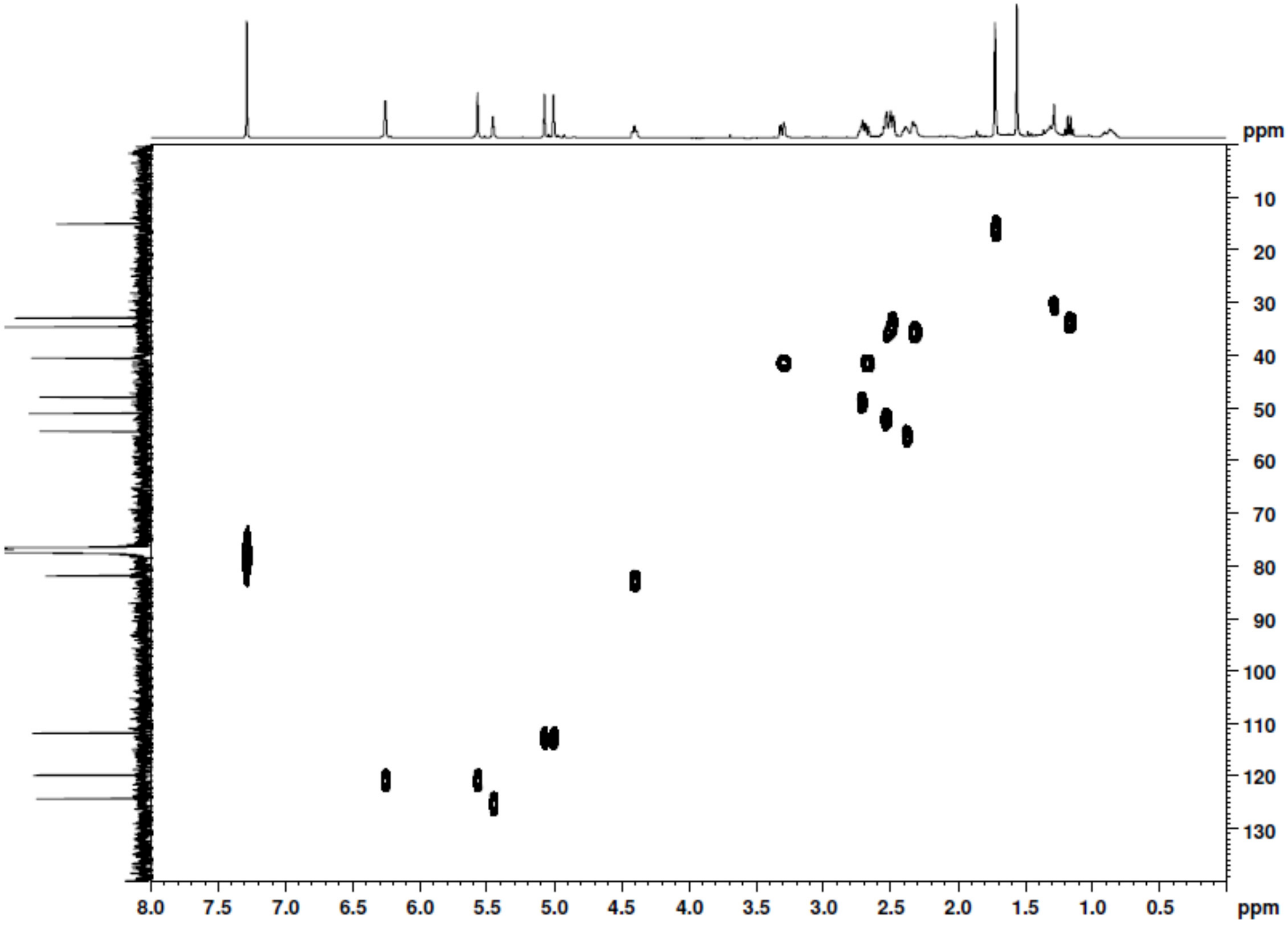


*S13.* HSQC spectrum of centaurolide B (**2**) (400 MHz, $CDCl_3$)

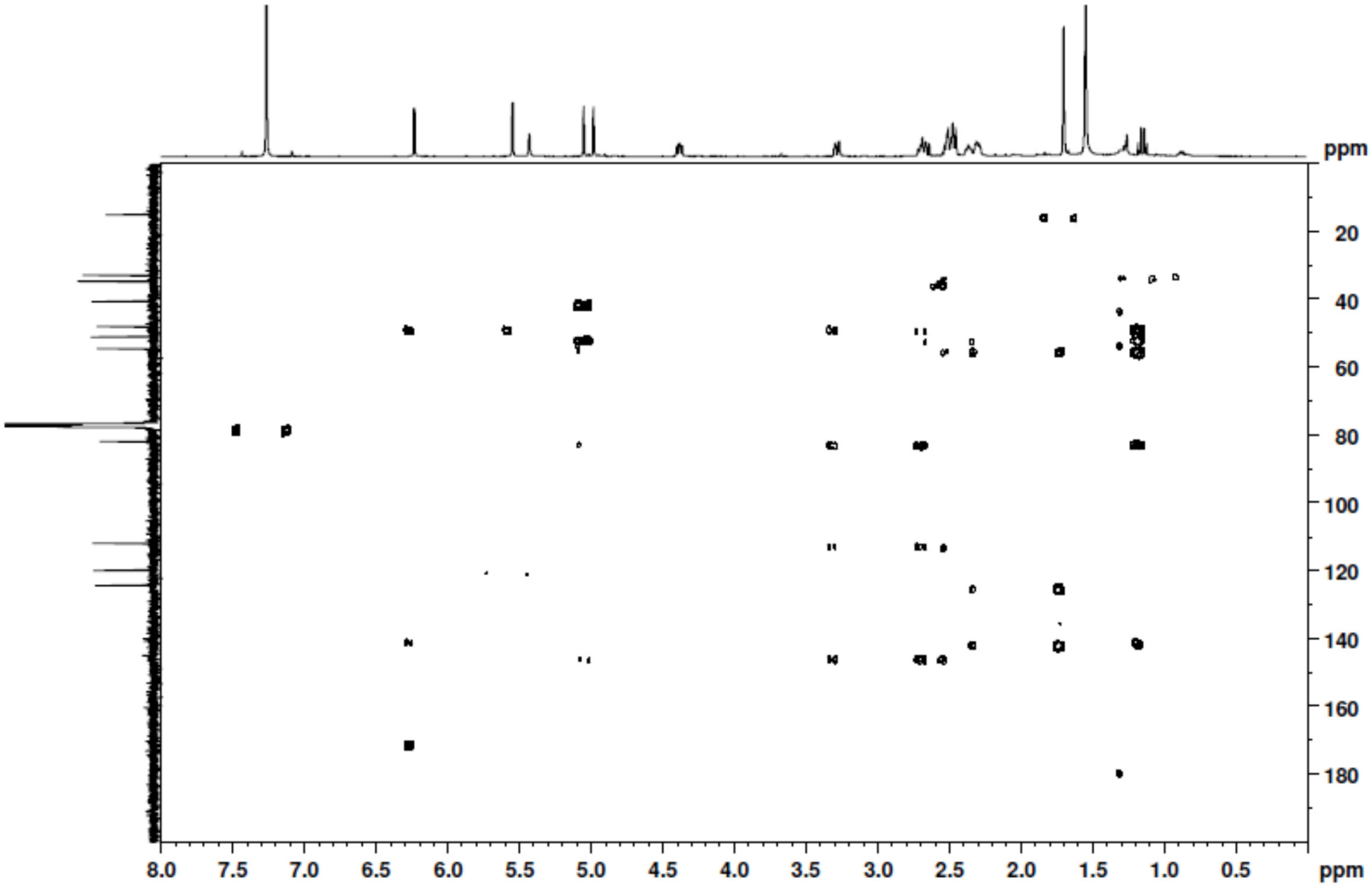


*S14*. HMBC spectrum of centaurolide B (**2**) (600 MHz, $CDCl_3$)

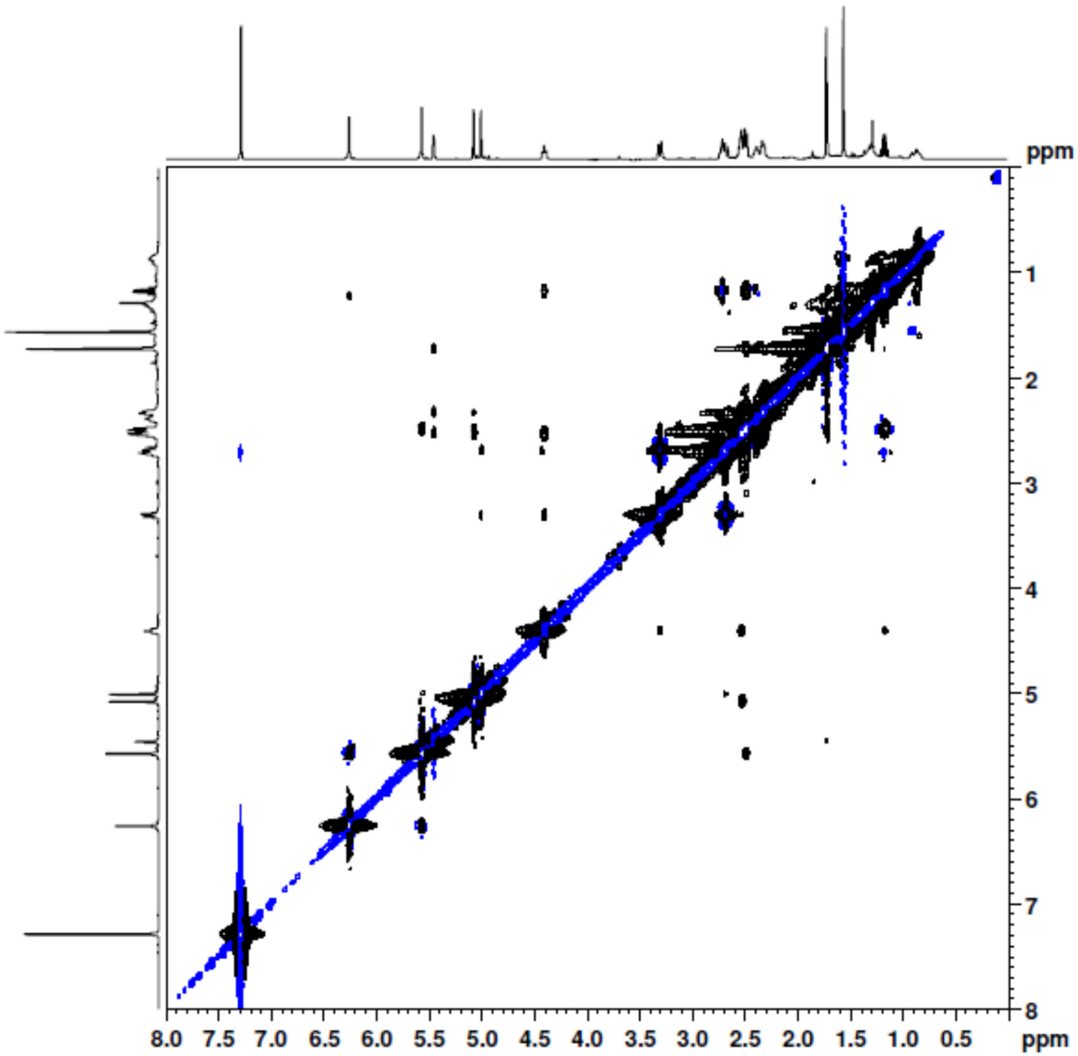


*S15*. NOESY spectrum of centaurolide B (**2**) (600 MHz, $CDCl_3$)

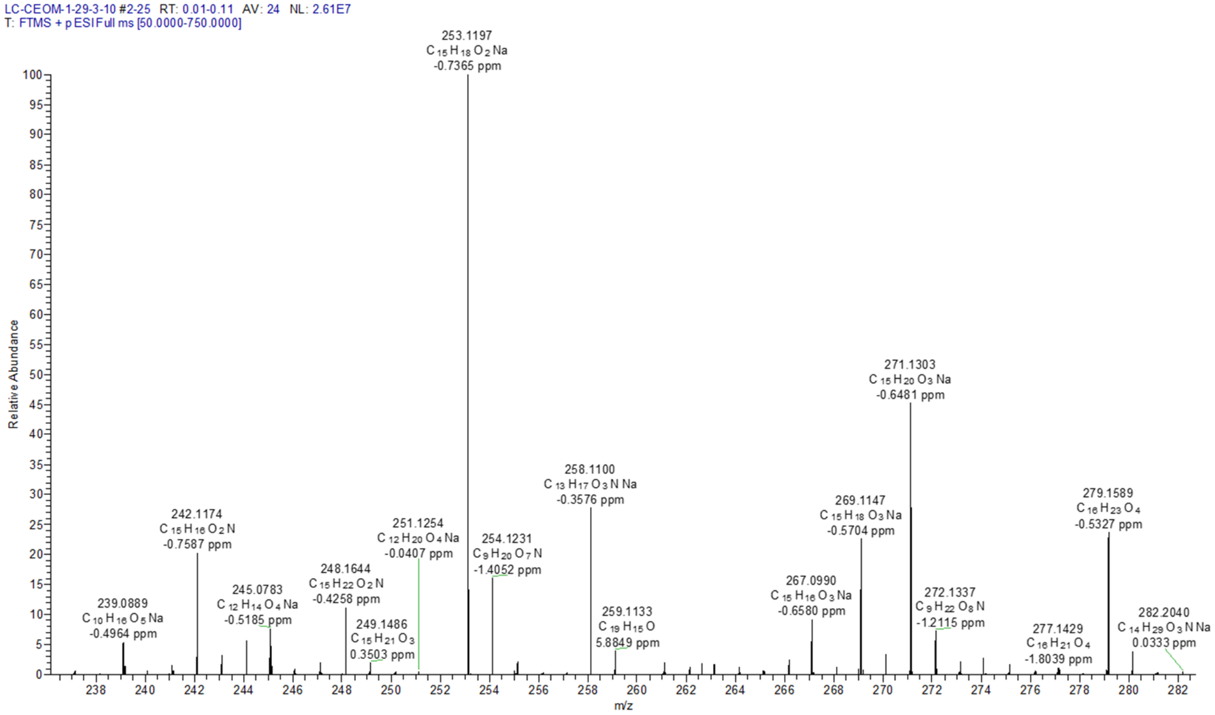


*S16*. HR ESIMS spectrum of centaurolide B (**2**)

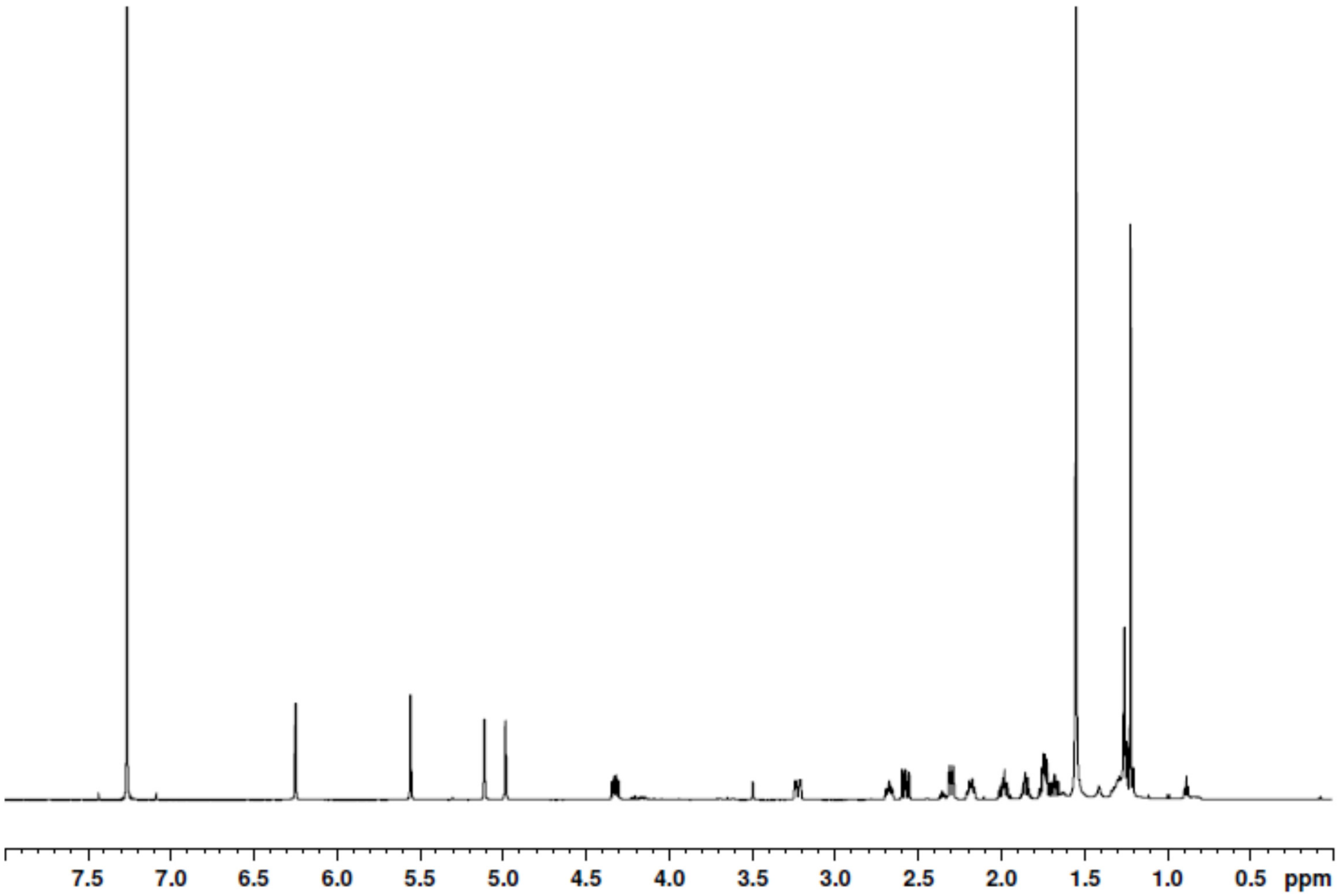


*S17*. $^{1}$H NMR spectrum of inuviscolide (**3**) (600 MHz, $CDCl_3$)

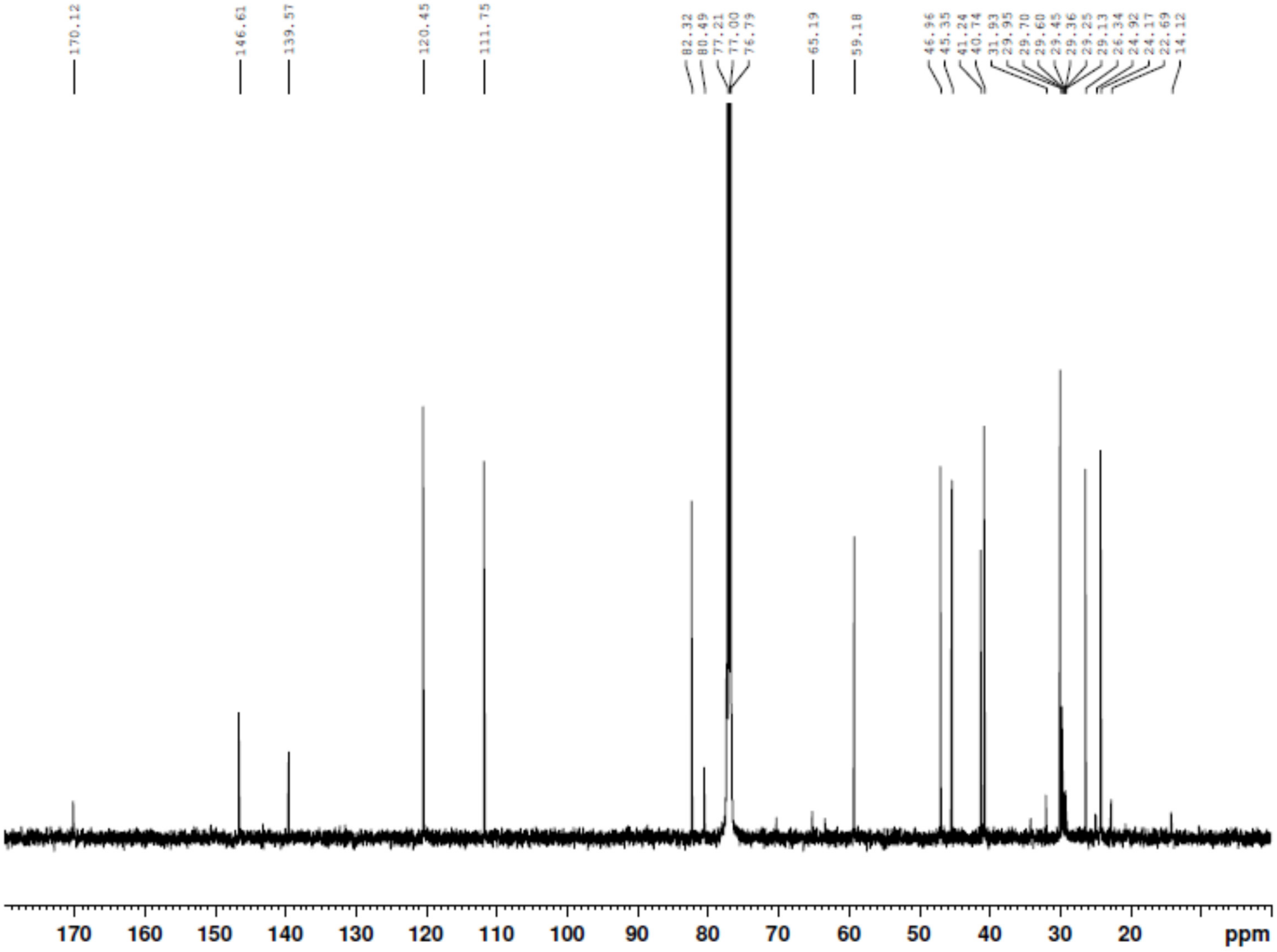


*S18.* $^{13}$C NMR spectrum of inuviscolide (**3**) (150 MHz, $CDCl_3$)

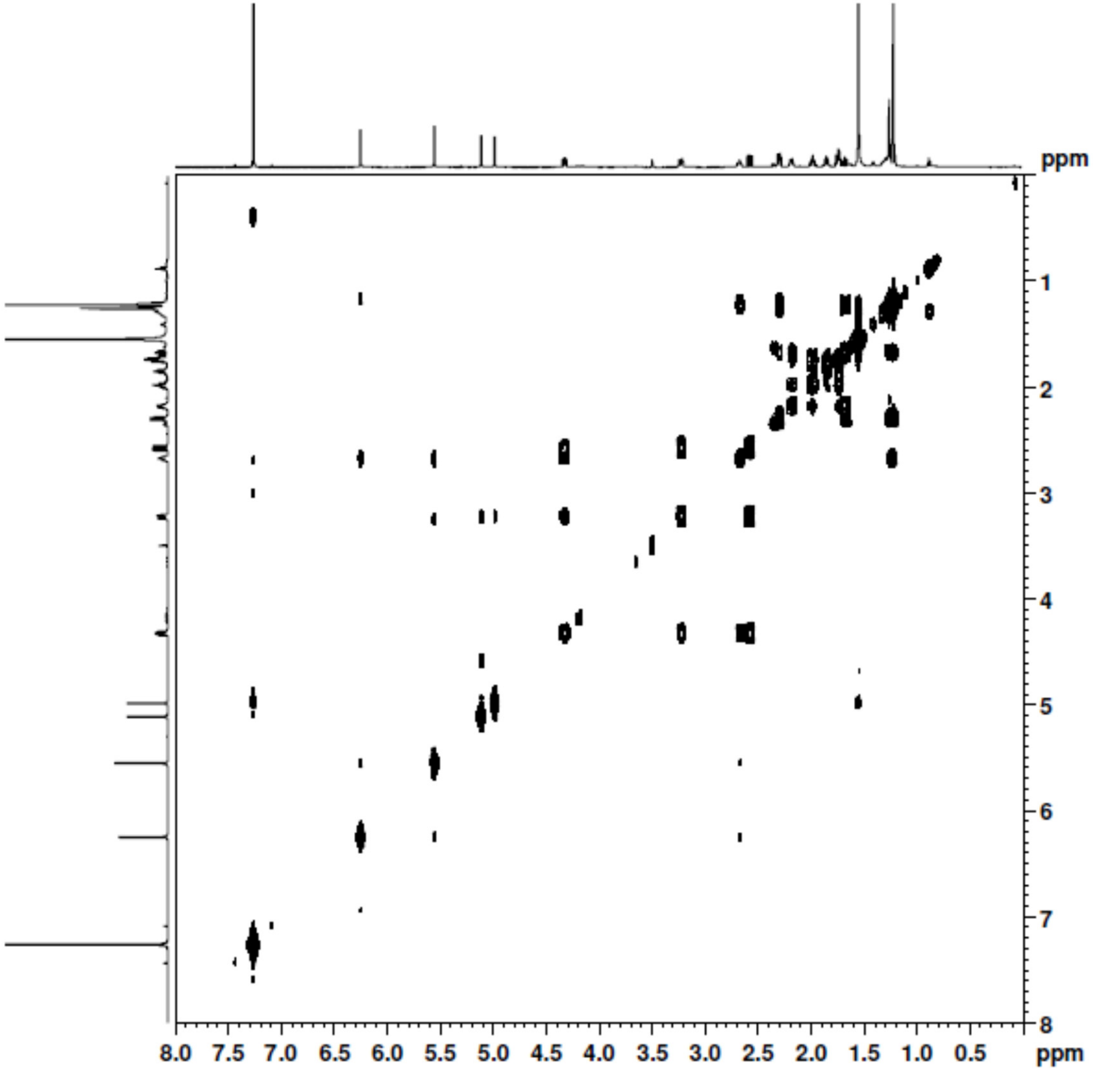


*S19.* COSY spectrum of inuviscolide (**3**) (600 MHz, $CDCl_3$)

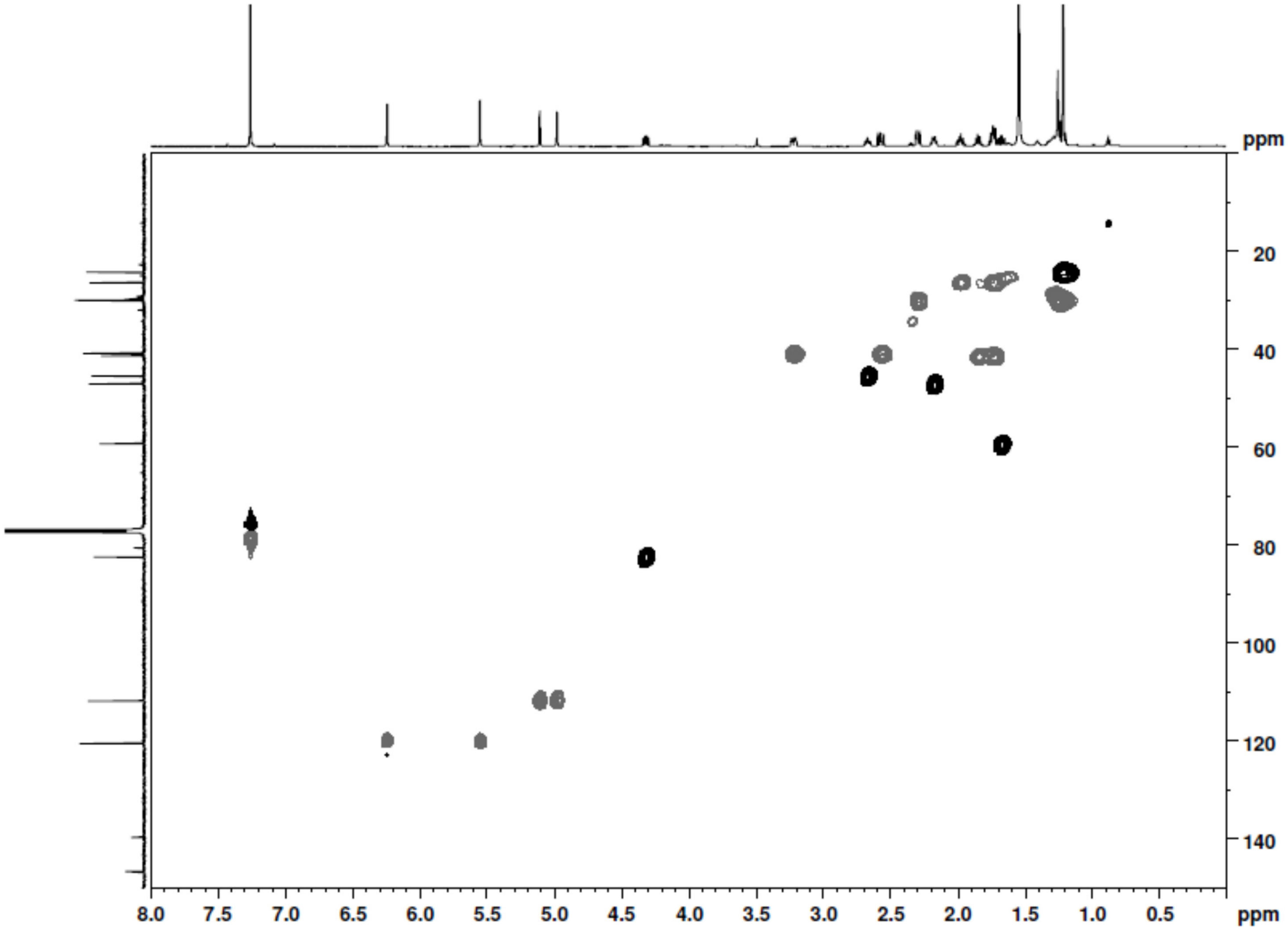


*S20.* ed-HSQC spectrum of inuviscolide (**3**) (600 MHz, CDCl3)

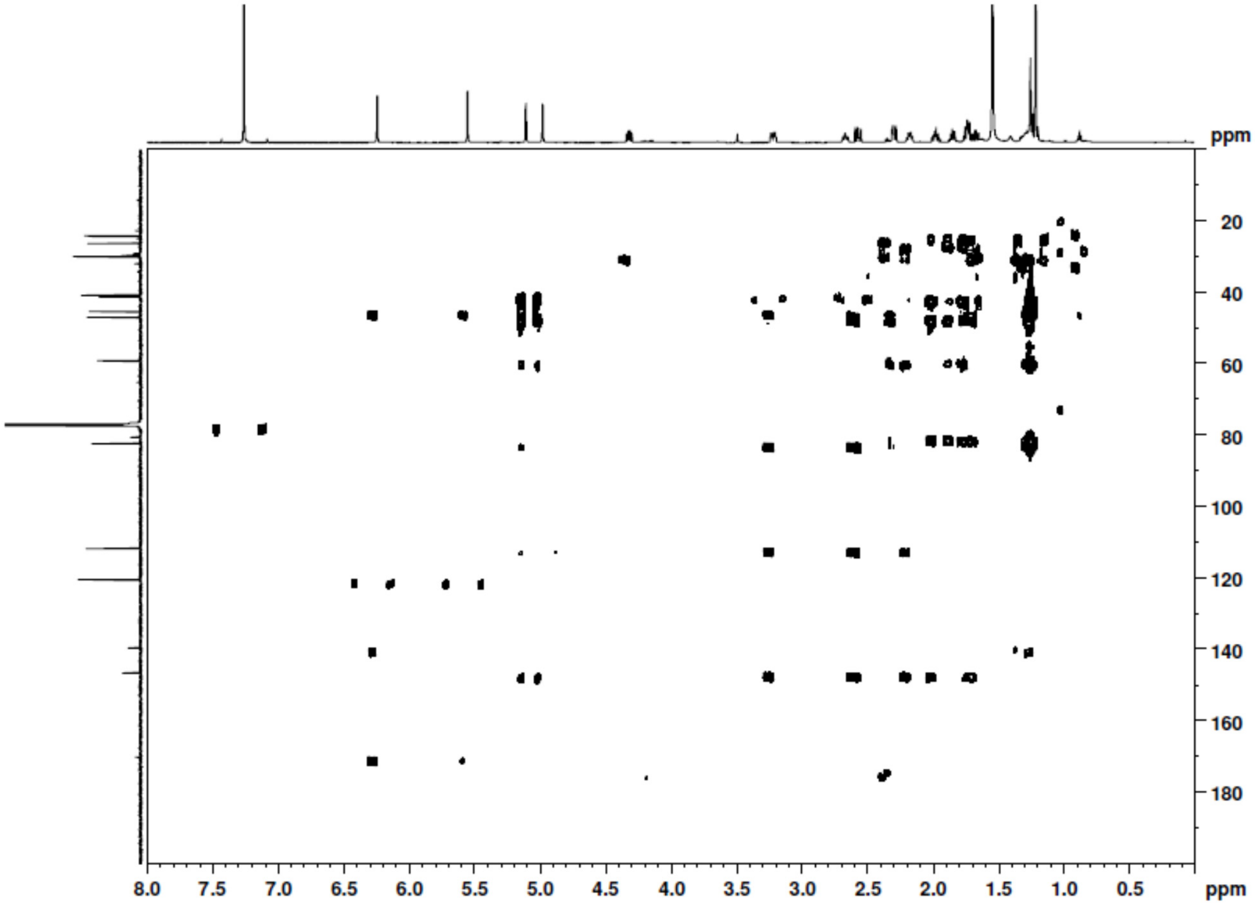


*S21.* HMBC spectrum of inuviscolide (**3**) (600 MHz, CDCl3)

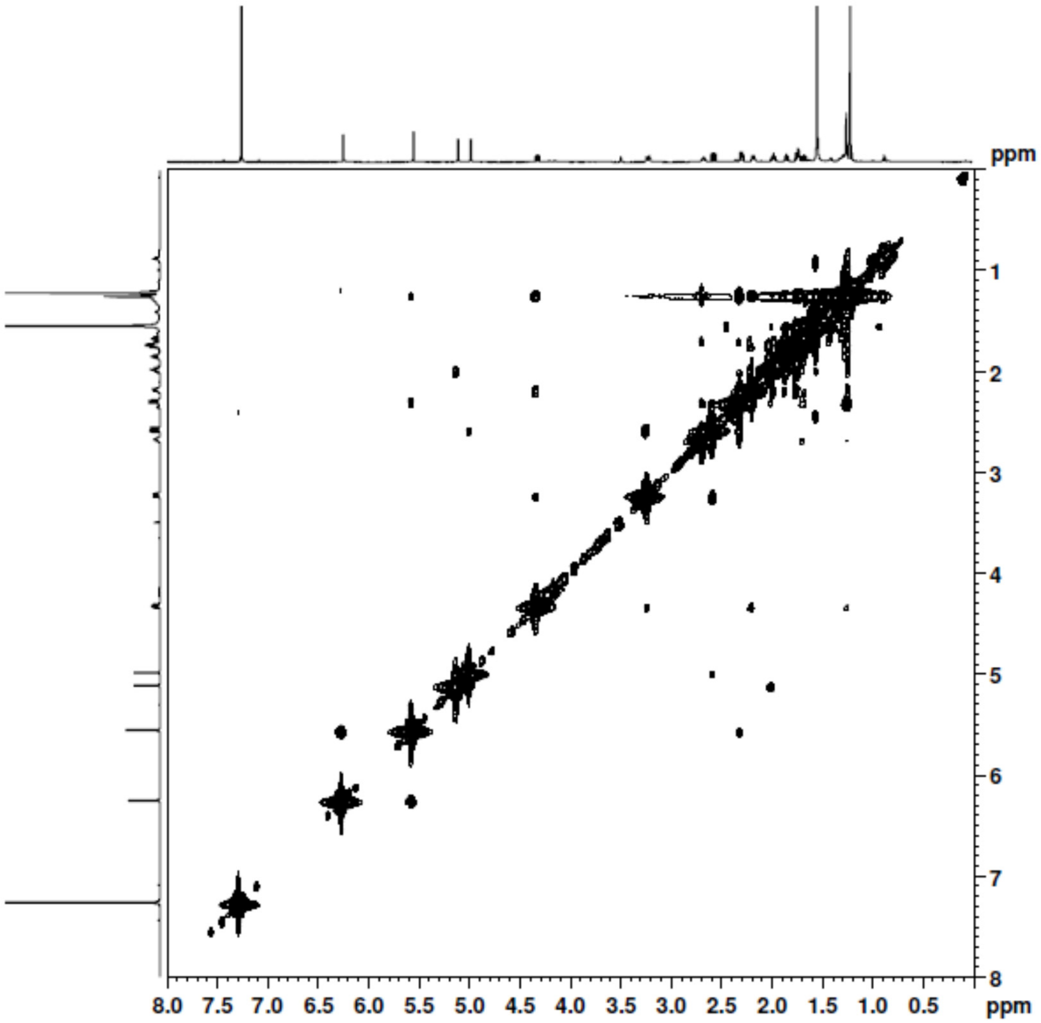


*S22*. NOESY spectrum of inuviscolide (**3**) (600 MHz, $CDCl_3$)

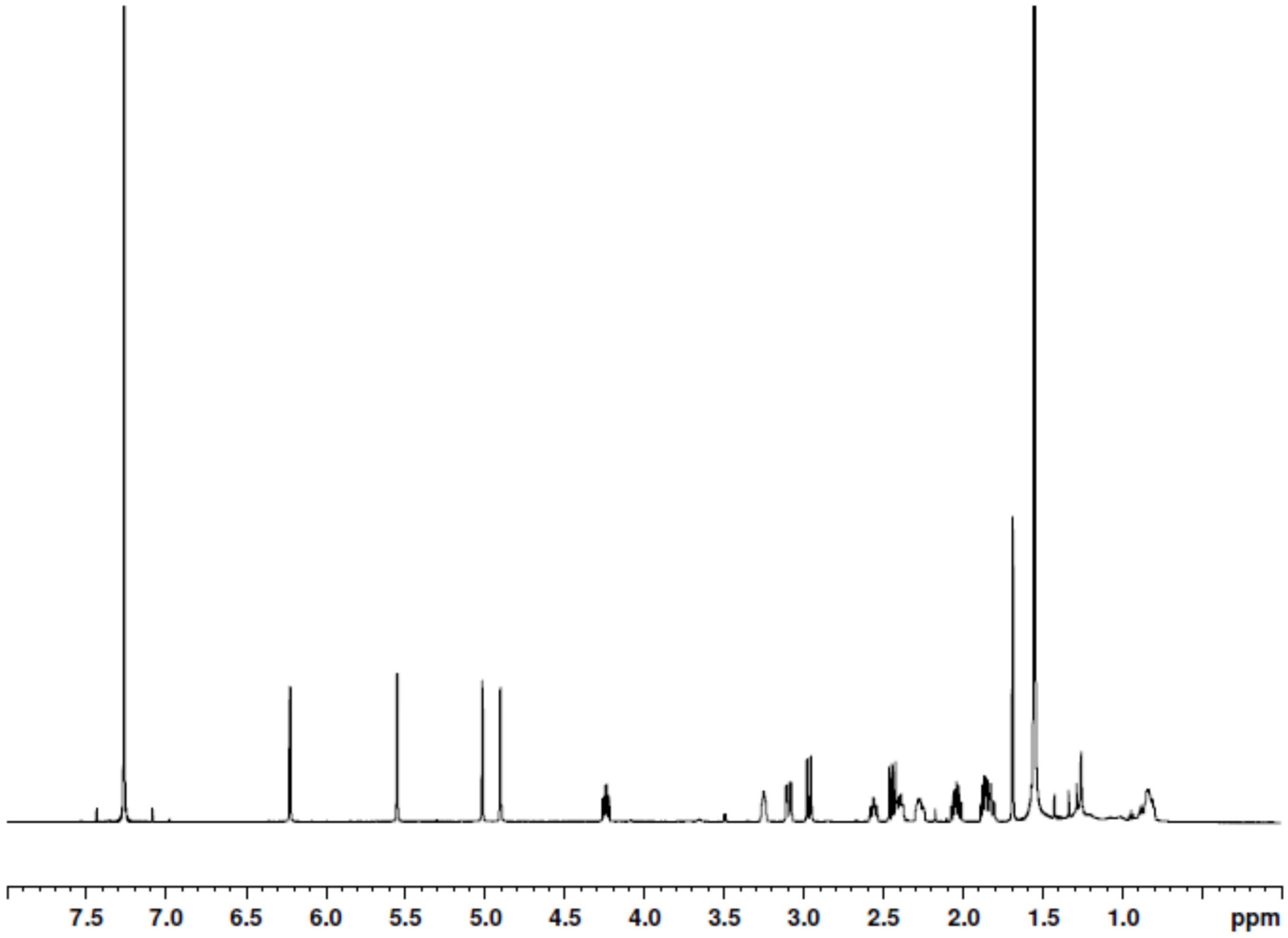


*S23.* $^{1}$H NMR spectrum of dehydration derivative of inuviscolide (600 MHz, $CDCl_3$)

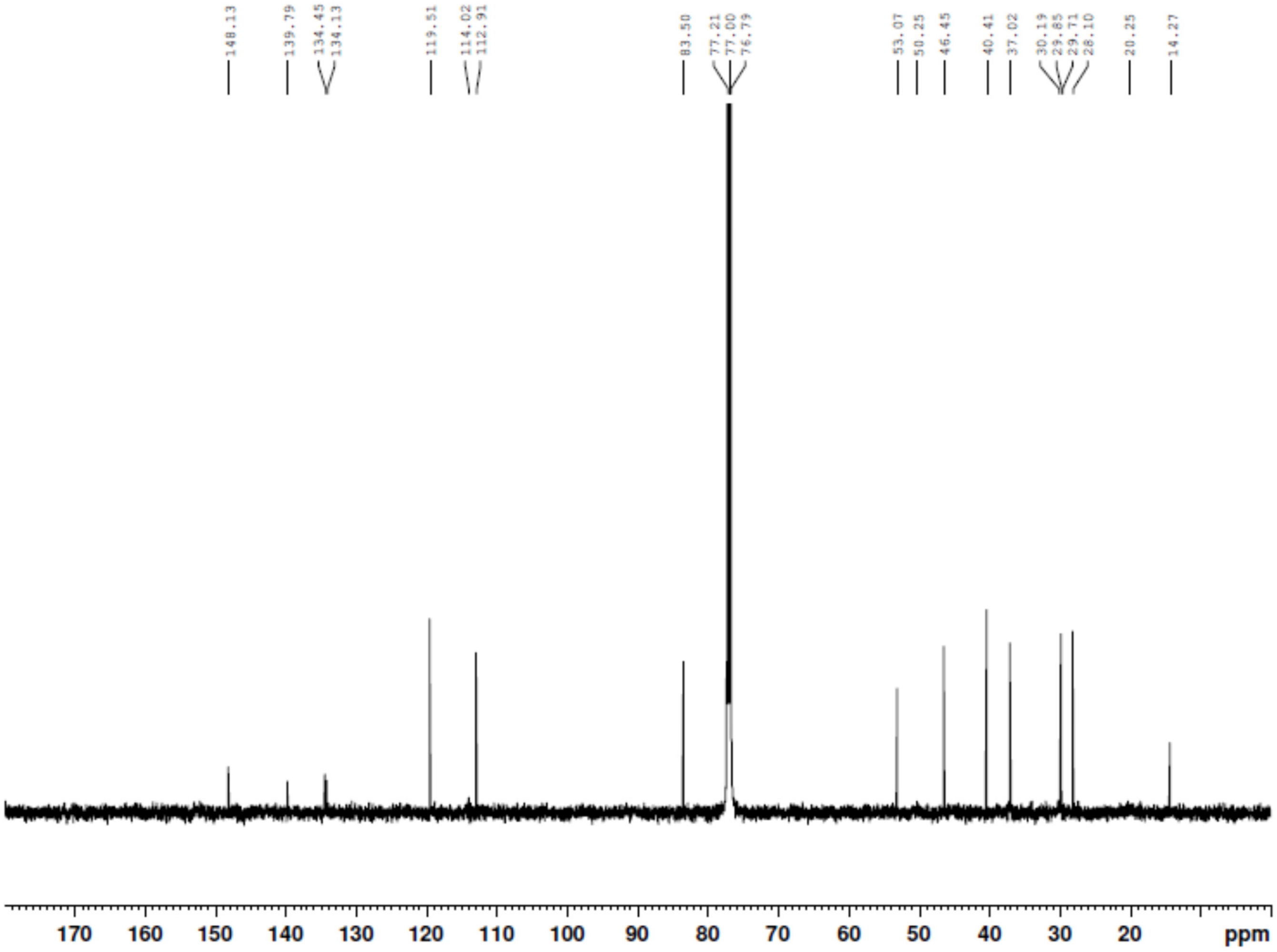


*S24*. $^{13}$C NMR spectrum of dehydration derivative of inuviscolide (600 MHz, $CDCl_3$)

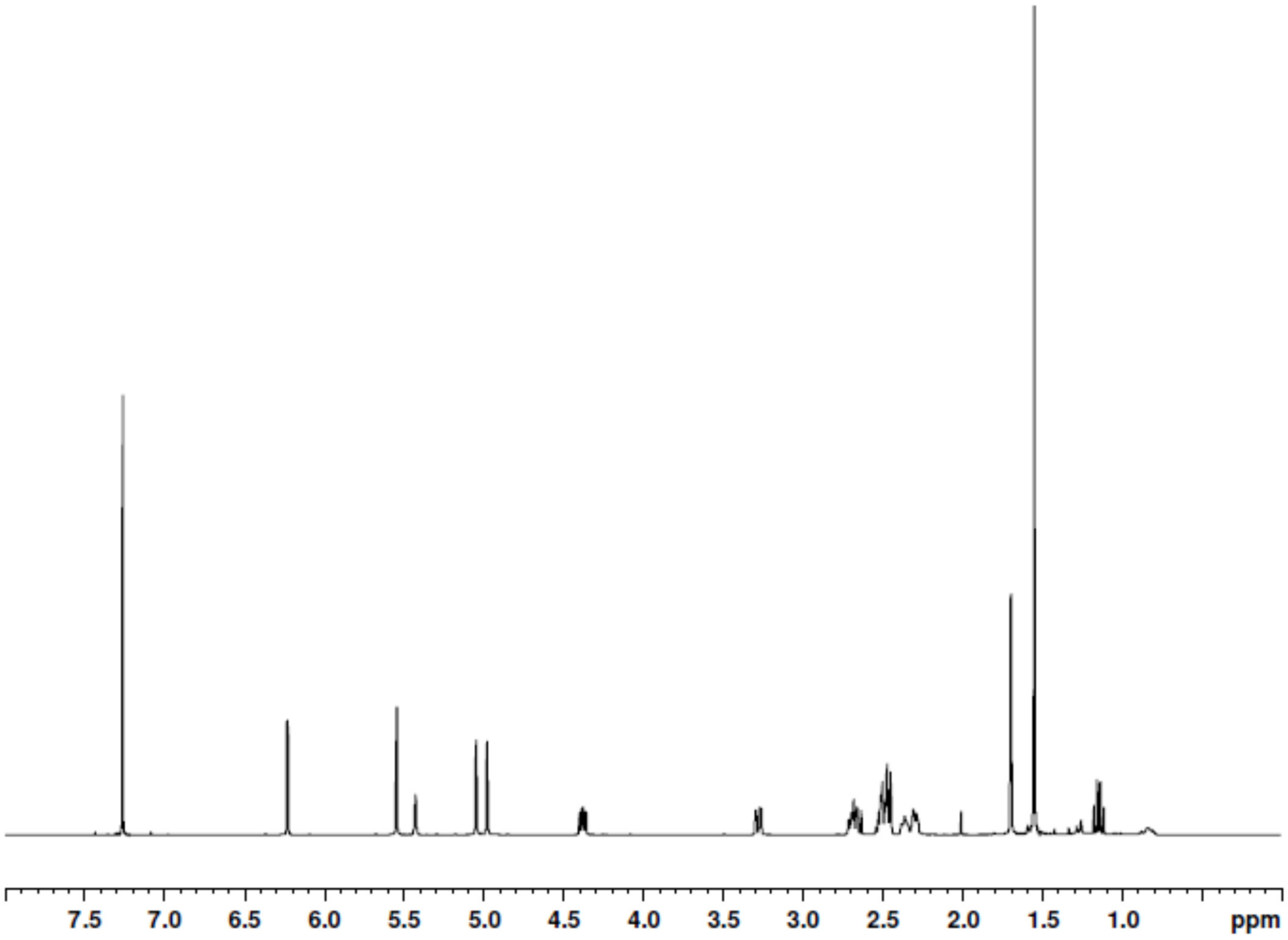


*S25*. $^{1}$H NMR spectrum of dehydration derivative of inuviscolide (600 MHz, $CDCl_3$)

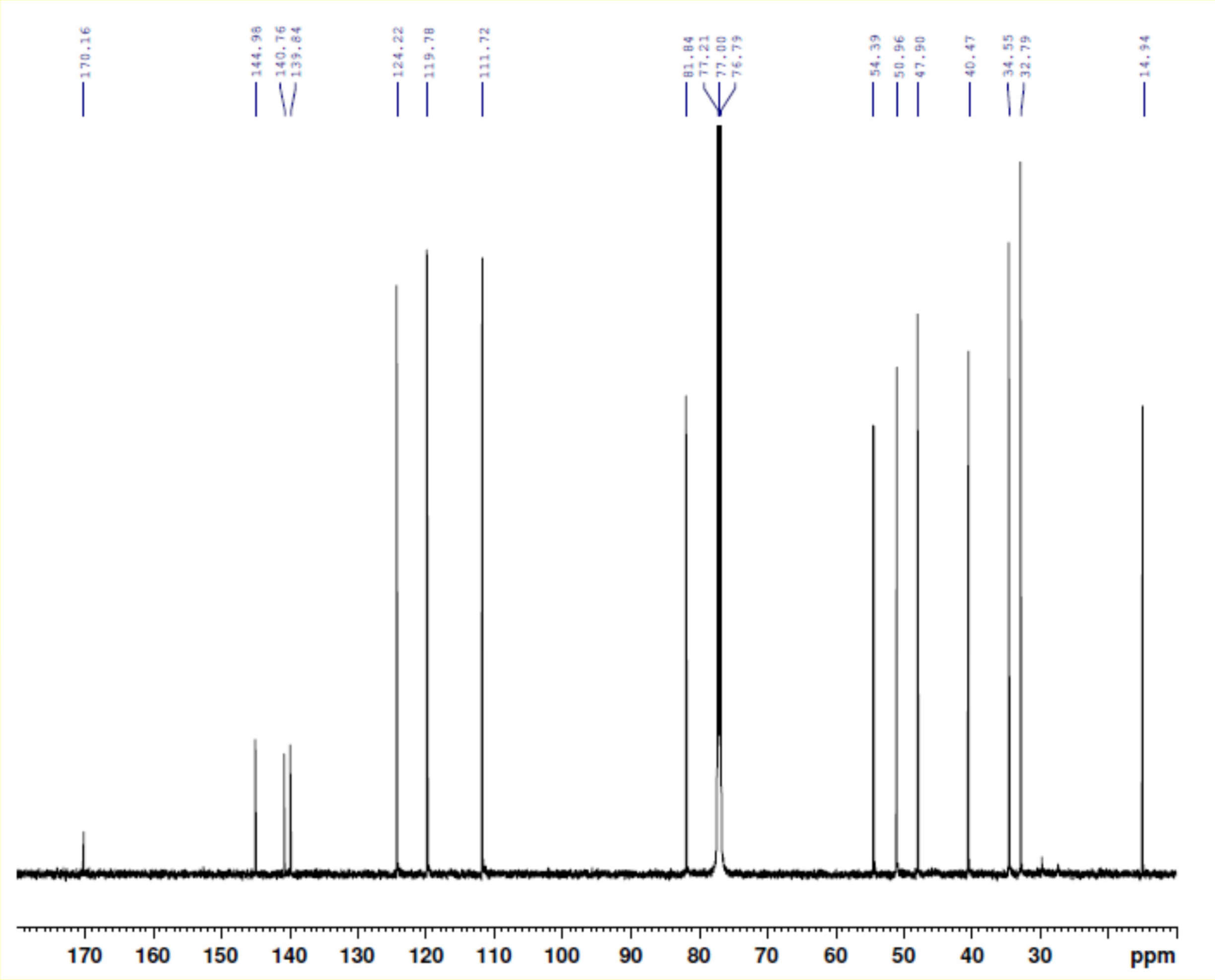


*S26*. $^{13}$C NMR spectrum of dehydration derivative of inuviscolide (600 MHz, $CDCl_3$)

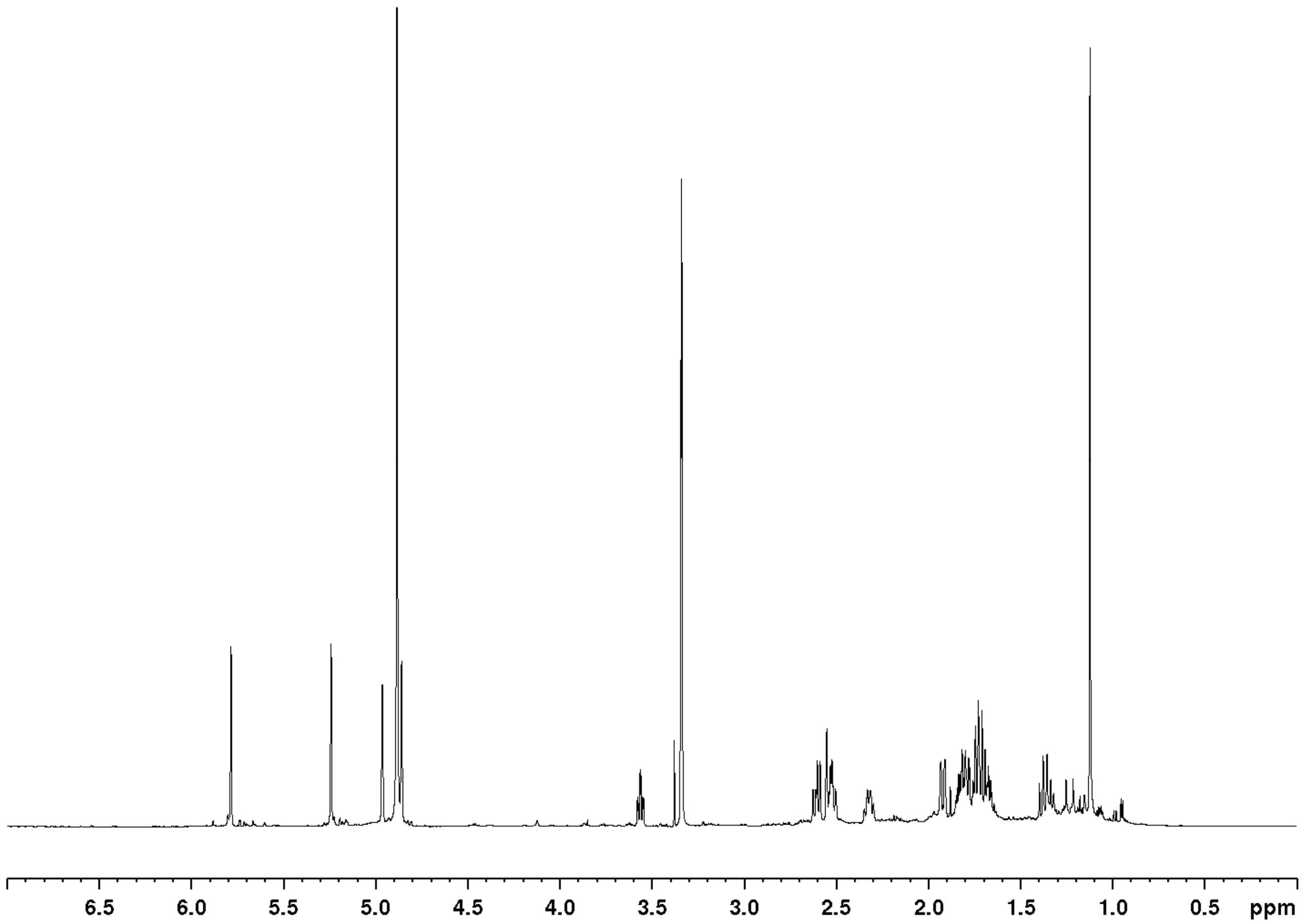


*S27*. $^1$H NMR spectrum of alcohol (**4**) (600 MHz, $CDCl_3$)

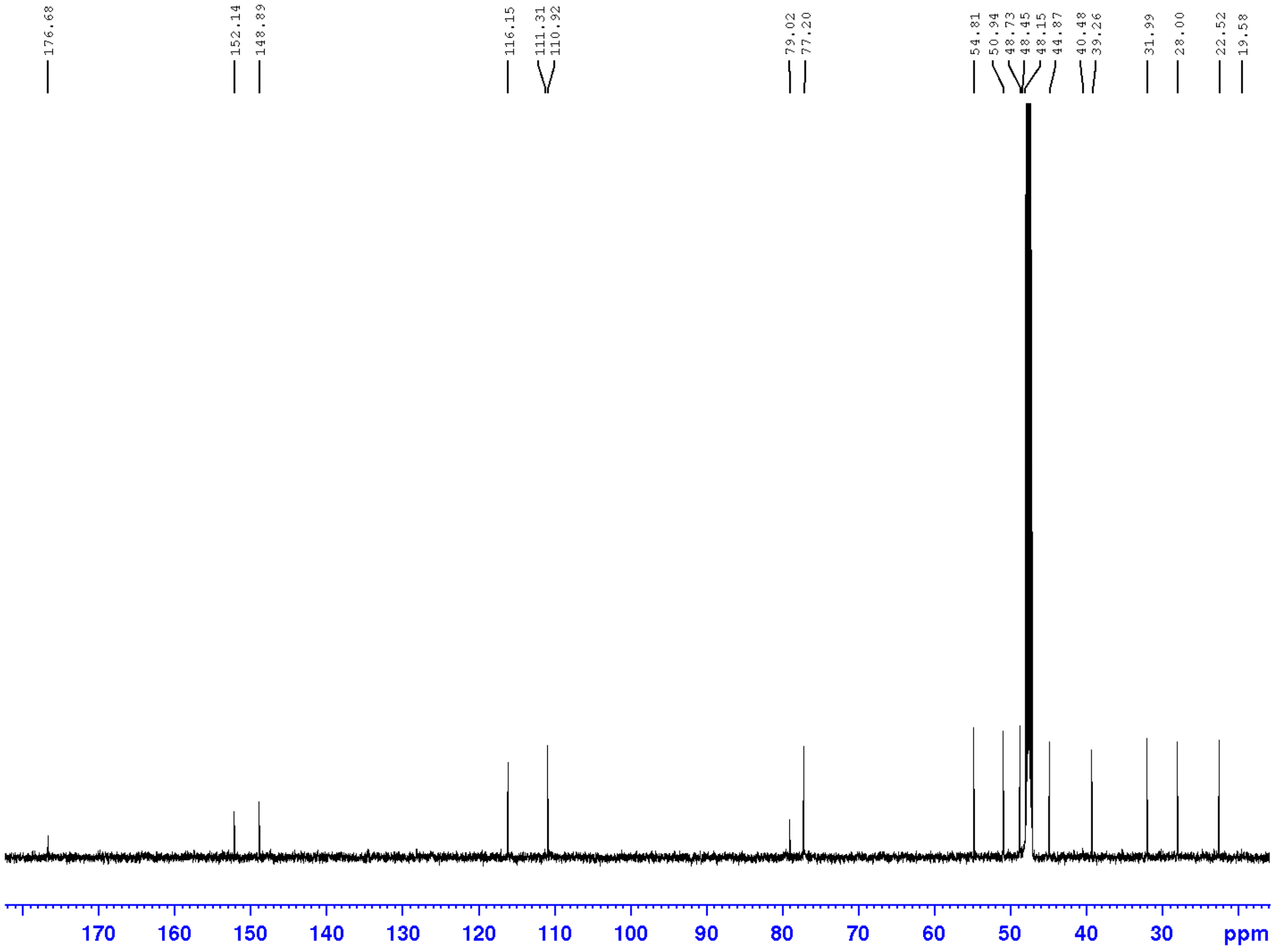


*S28.* $^{13}$C NMR spectrum of alcohol (**4**) (150 MHz, $CDCl_3$)

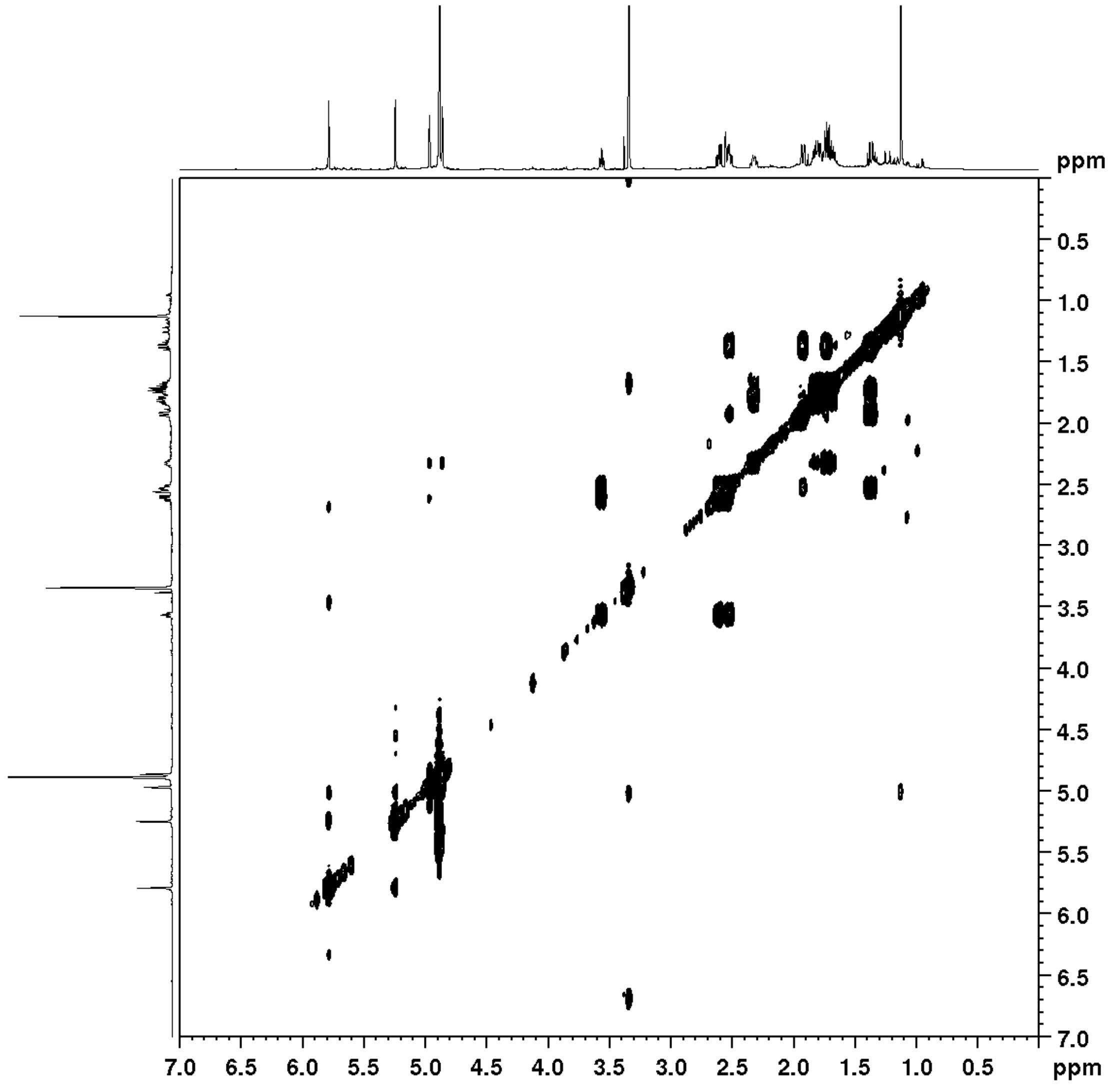


*S29*. COSY spectrum of alcohol (**4**) (600 MHz, $CDCl_3$)

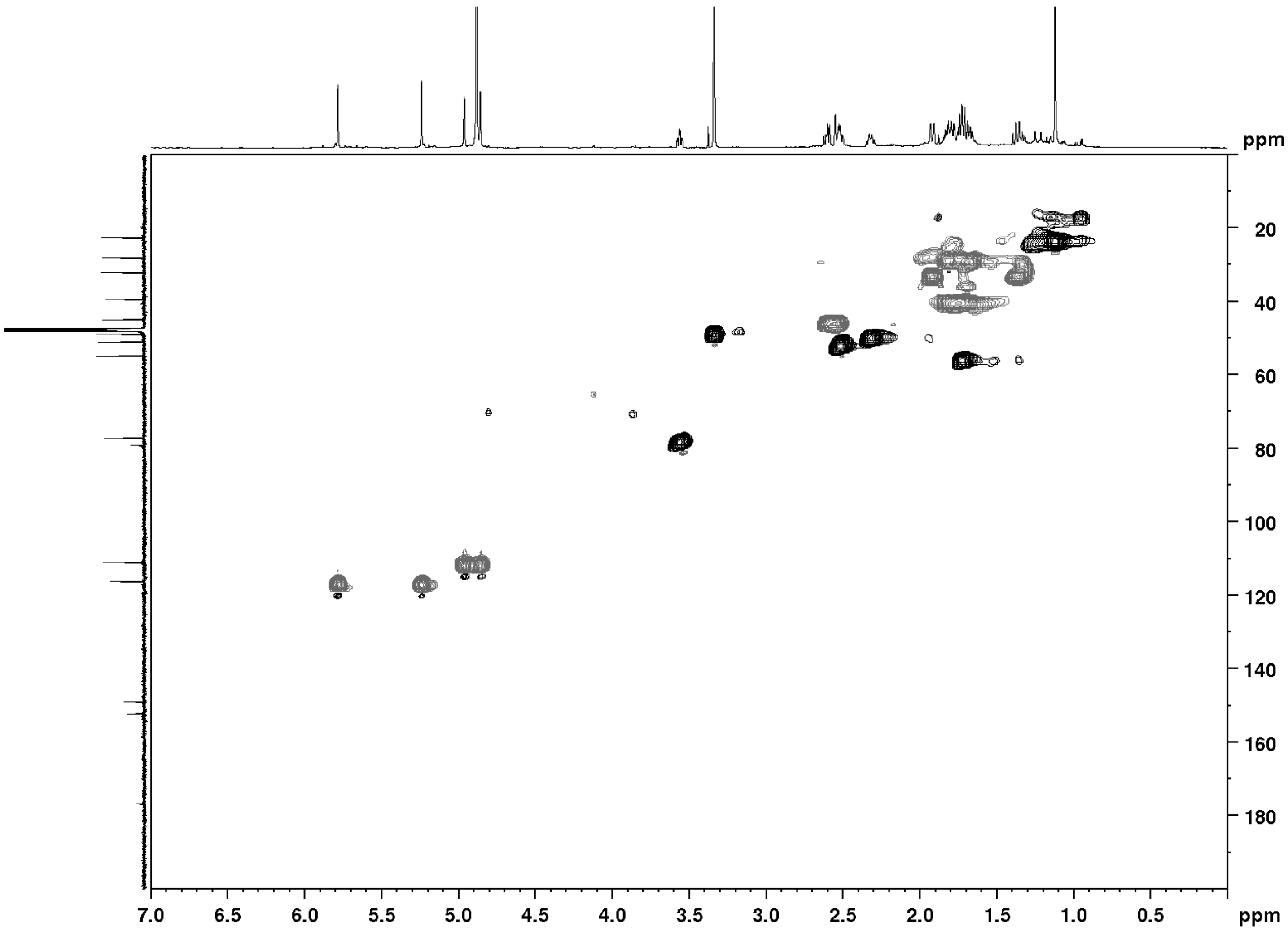


*S30.* ed-HSQC spectrum of alcohol (**4**) (600 MHz, $CDCl_3$)

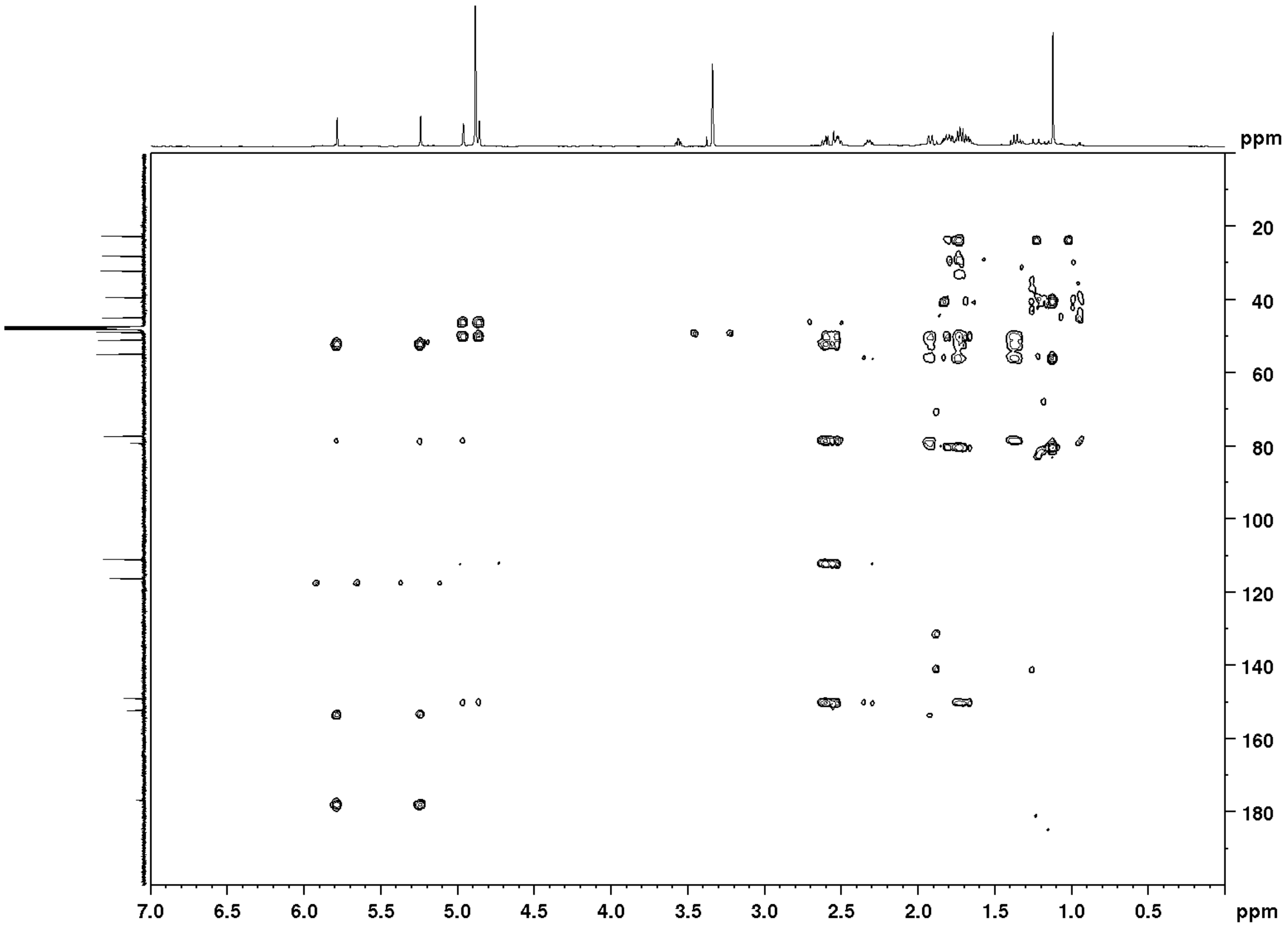


*S31.* HMBC spectrum of alcohol (**4**) (600 MHz, CDCl3)

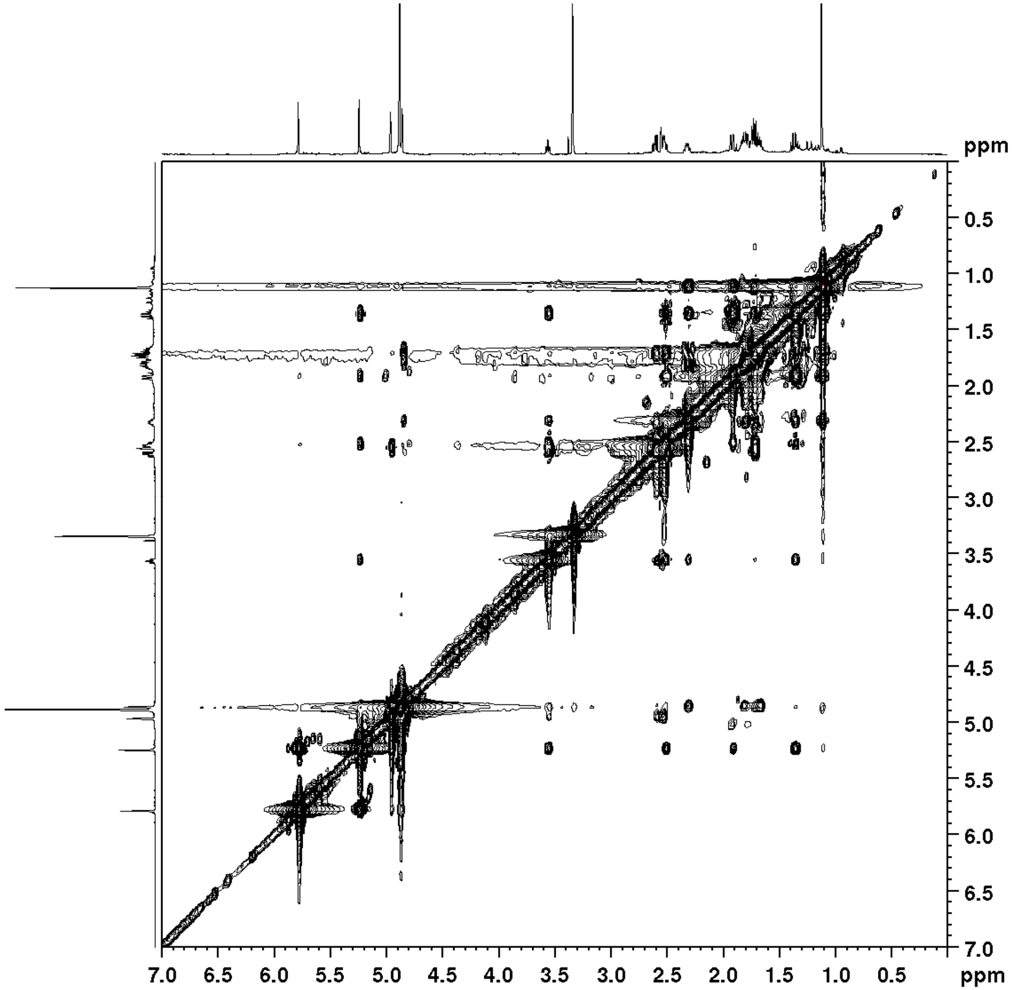


*S32*. NOESY spectrum of alcohol (**4**) (600 MHz, $CDCl_3$)
